# Prokaryotic, Fungal, and Unicellular Eukaryotic Core Communities Across Three Sympatric Marine Sponges From the Southwestern Atlantic Coast Are Dominated Largely by Deterministic Assemblage Processes

**DOI:** 10.3389/fmicb.2021.674004

**Published:** 2021-06-08

**Authors:** Cristiane C. P. Hardoim, Gisele Lôbo-Hajdu, Márcio R. Custódio, Pablo R. Hardoim

**Affiliations:** ^1^Institute of Biosciences, São Paulo State University, São Paulo, Brazil; ^2^Department of Genetic, Biology Institute Roberto Alcântara Gomes, Rio de Janeiro State University, Rio de Janeiro, Brazil; ^3^Department of Physiology, Biosciences Institute and NP-Biomar, Center for Marine Biology, University of São Paulo, São Paulo, Brazil; ^4^Department of R&D, Biopromo, São Paulo, Brazil

**Keywords:** microbial community assembly, stochastic process, deterministic process, neutral community model, Brazilian coast, co-evolution, Porifera holobiome

## Abstract

Marine sponges are known to harbor a diverse and complex microbiota; however, a vast majority of surveys have been investigating the prokaryotic communities in the north hemisphere and Australia. In addition, the mechanisms of microbial community assembly are poorly understood in this pivotal player of the ecosystem. Thus, this survey addressed the holobiome of the sponge species in the São Paulo region (Brazil) for the first time and investigated the contribution of neutral and niche processes of prokaryotic, fungal, and unicellular eukaryotic assemblage in three sympatric species *Aplysina caissara*, *Aplysina fulva*, and *Tedania ignis* along with environmental samples. The compositions of the holobiome associated with the sponges and detected in environmental samples were strikingly different. Remarkably, between 47 and 88% of the assigned operational taxonomic units (OTUs) were specifically associated with sponge species. Moreover, around 77, 69, and 53% of the unclassified OTUs from prokaryotic, fungal, and unicellular eukaryotic communities, respectively, showed less than 97% similarity with well-known databases, suggesting that sponges from the southwestern Atlantic coast are an important source of microbial novelty. These values are even higher, around 80 and 61% of the unclassified OTUs, when excluding low abundance samples from fungal and unicellular eukaryotic datasets, respectively. Host species were the major driver shaping the sponge-associated microbial community. Deterministic processes were primarily responsible for the assembly of microbial communities in all sponge species, while neutral processes of prokaryotic and fungal community assembly were also detected in the sympatric *A. caissara* and *T. ignis* replicates, respectively. Most of the species-rich sponge-associated lineages from this region are also found in the Northern seas and many of them might play essential roles in the symbioses, such as biosynthesis of secondary metabolites that exhibit antimicrobial and antiviral activities, as well as provide protection against host predation. Overall, in this study the microbiota was assembled by interactions with the host sponge in a deterministic-based manner; closely related sponge species shared a strong phylogenetic signal in their associated prokaryotic and fungal community traits and Brazilian sponges were a reservoir of novel microbial species.

## Introduction

Marine sponges harbor abundant, diverse, and complex microbiomes, which include bacteria and archaea (Taylor et al., 2007; Thomas et al., 2016). Based on the abundance, these animals have been classified into high microbe abundance (HMA) and low microbe abundance (LMA) sponges (Hentschel et al., 2003). The diversity of bacteria associated with sponges has been extensively investigated followed by archaea (Taylor et al., 2007; Hardoim et al., 2012; Hentschel et al., 2012; Webster and Taylor, 2012; Thomas et al., 2016). The prokaryotic community from 268 sponge species collected from several locations encompassed 60 to 72 recognized and candidate phyla (Moitinho-Silva et al., 2017a). A previous prokaryotic survey of three sympatric marine sponges from the southwestern Atlantic coast of Brazil (São Paulo state) revealed a total of 51 associated phyla (Hardoim et al., 2021). Among them, Nanoarchaeota, Elusimicrobiota, Parcubacteria, and others were detected for the first time. Moreover, a high degree of novel prokaryotic diversity was also associated with these marine sponges.

Much less attention has been given to microbial eukaryotic communities associated with marine sponges, especially regarding their ecology and function with their hosts (Taylor et al., 2007; Webster and Taylor, 2012; Thomas et al., 2016). To date, very few studies have applied high-throughput sequencing technology to study both fungal and other microbial eukaryotic communities associated with marine sponges. The fungi associated with sponges were considered to have high host-specificity (De Mares et al., 2017), low host-specificity (Nguyen and Thomas, 2018), or no specificity at all (Naim et al., 2017). For fungal assemblages associated with sponges, two genes have been routinely used, the 18S rRNA gene and internal transcribed spacer (ITS) of the ribosomal RNA gene operon (De Mares et al., 2017; Naim et al., 2017; Nguyen and Thomas, 2018). Overall, the associated fungi are phylogenetically diverse and both Ascomycota and Basidiomycota were the dominant phyla (De Mares et al., 2017; Naim et al., 2017; Nguyen and Thomas, 2018).

The unicellular eukaryotic community associated with marine sponges is largely unknown. Recently, this community was assessed with 18S rRNA gene metabarcoding analysis in sympatric species and showed no host-specificity (De Mares et al., 2017). Besides, there was no significant difference between eukaryotic communities detected in seawater and those associated with the sponge hosts, indicating no selection by the host. Overall, members of 94 eukaryotic phyla and approximate phylum-level groups were detected in sponges and seawater (De Mares et al., 2017). This result suggests that, although important roles are played by the unicellular eukaryotic community for ecosystem functioning, the assembly of these communities are dominated by stochastic processes.

The mechanisms of the community assembly responsible for shaping the structure of the sponge microbiomes continue to remain uncertain. Hosts and their microbiomes are ecological systems structured by multitrophic interactions governed by deterministic and stochastic processes (Miller et al., 2018). Deterministic processes assume that species traits, interspecies interactions such as mutualism, competition, predation, and trade-offs, environmental factors such as nutrient heterogeneity, pH, temperature, moisture, and salinity, and host environments shape the microbial community structure (i.e., species composition and abundance distributions; Vellend, 2010; Zhou and Ning, 2017). On the other hand, stochastic processes, such as birth, death, immigration and emigration, spatiotemporal variation, and/or historical contingency also play a role in community assembly (Chen et al., 2017; Zhou and Ning, 2017). Both processes are recognized as occurring simultaneously in shaping the assembly of microbial communities (Gravel et al., 2006; Chase and Myers, 2011; Stegen et al., 2016). Therefore, each ecological process of selection, dispersal, diversification, and drift might affect the microbial species on its own terms. Furthermore, studies have shown that microbial species can be partitioned into habitat generalists and specialists, on the basis of their distinct capacities to respond to environmental challenges (Pandit et al., 2009; Székely and Langenheder, 2014). Often, habitat generalists have broad environmental tolerances and can occur in many habitats, whereas habitat specialists are more restricted in sites, due to their narrow environmental tolerances (Pandit et al., 2009). Hence, habitat generalists and specialists might respond distinctively under changing environmental conditions. For the sponge microbiome, the influences of deterministic and stochastic processes affecting the distribution of habitat generalists and specialists remain unknown.

The microbial diversity and community composition of the three domains of life have rarely been assessed for the same host sponges and, to the best of our knowledge, have never been assessed for southwestern Atlantic species. Thus, in the present study, the bacteria, archaea, fungi, and unicellular eukaryotic communities associated with *Aplysina caissara*, *Aplysina fulva*, and *Tedania ignis* were investigated. *Aplysina caissara* is so far considered endemic to the southern and southeastern Brazilian coast (Pinheiro and Hajdu, 2001; Pinheiro et al., 2007; Van Soest, 2017). On the other hand, *A. fulva* and *T. ignis* are widespread on the Brazilian coast as well as in the Caribbean Sea (Pinheiro and Hajdu, 2001; Pinheiro et al., 2007; Van Soest et al., 2021). The genus *Aplysina* imposes a challenge for identification due to the lack of morphological features (Pinheiro et al., 2007). To aid species identification, sponge DNA barcoding was employed (Wörheide and Erpenbeck, 2007). The three sponge species, seawater, and sediment samples were collected along the north coast of São Paulo state (São Sebastião, Brazil). This region is characterized by the transition between tropical and temperate southwestern Atlantic marine ecoregions (Spalding et al., 2007) and encompasses over 70 sponge species belonging to the classes Calcarea and Demospongiae (Custódio and Hajdu, 2011). It could be considered a local hotspot of sponge diversity in Brazil. Our survey sought to test the following hypotheses: (*i*) sponge species exhibit host-specificity for the three domains of life, (*ii*) the sponge microbiome shows co-evolution with the host sponge, (*iii*) both deterministic and stochastic processes contribute to the microbiota community assembly associated with sponge species, and (*iv*) habitat specialists are largely assembled by HMA sponges as compared to habitat generalists. This study aims to investigate beyond the descriptive understanding of the sponge-associated microbiota by developing predictive microbial assembly interpretations.

## Materials and Methods

### Sponge and Environmental Sampling

Samples were collected at Praia Preta, São Sebastião (23°49' 24.24'' S–45°24' 40.679'' W) along the coast of São Paulo state, Brazil, tropical southwestern Atlantic, in March 2019. Measurements of salinity and temperature at the time of sampling were 32.62 parts per million and 28.7°C, respectively. Sampling occurred in two dives around 20 min apart from one another. In the first dive, 10 surrounding seawater samples of 1 L each were collected from about 1 m in the vicinity of the sponge specimens and placed in sterile plastic bottles. Five sediment samples of 2 kg each were also collected and placed in sterile Ziplock bags. In the second dive, five specimens each of the abundant and sympatric *Aplysina caissara* (Pinheiro and Hajdu, 2001; Demospongiae, Verongiida, Aplysinidae), *Aplysina fulva* (Pallas, 1766; Demospongiae, Verongiida, Aplysinidae), and *Tedania ignis* (Duchassaing de Fonbressin and Michelotti, 1864; Demospongiae, Poecilosclerida, Tedaniidae) were collected by scuba diving at depths of around 2.5 m and placed separately *in situ* in sterile Ziplock bags containing natural seawater. *In situ* pictures of the specimens were taken to aid identification. All samples were placed in cooling boxes, transported to the laboratory (*c*. 10 min) at the Centre for Marine Biology of the São Paulo University (CEBIMar/USP) for initial processing. Environmental samples were maintained at −20°C until further use. Prior to sample processing, the sponge specimens were rinsed with sterile artificial seawater (ASW; McLachlan, 1964) to remove loosely associated organisms. Voucher samples were preserved in 70% ethanol for classical taxonomic identification. Pieces from the inner part of the sponge specimens were preserved in RNALater (QIAGEN, Hilden, Germany) at 4°C overnight and then transferred to −20°C.

### Total Community DNA Extraction

All genomic DNA was extracted using a DNeasy PowerSoil DNA isolation kit (QIAGEN, Hilden, Germany) according to the manufacturer’s protocol. Five seawater samples (1 L) were filtered through 0.22 μm pore-size nitrocellulose filters to assess the prokaryotic community, whereas the other five samples were filtered through 0.45 μm pore-size nitrocellulose filters to assess the fungal and unicellular eukaryotic communities (Merck Millipore, Burlington, MA, United States) using a vacuum pump. The filters were then cut into small pieces and directly used for DNA extraction. Sediment samples were mixed, sieved, and an aliquot of 0.25 g was used for DNA extraction. To assess sponge microbial communities, about 0.25 g of internal sponge body was used for DNA extraction.

### Sponge Barcoding

Classical identification of the sponges was accomplished using standard methods: spicule and skeletal preparations followed by Hajdu et al. (2011) and spongin fibers were prepared according to Pinheiro and Hajdu (2001). Additionally, the sponge DNA barcoding (Wörheide and Erpenbeck, 2007) was performed as explained in detail in Hardoim et al. (2021). To investigate the capacity of the cytochrome b (cob) gene to separate *Aplysina* species, vouchers representatives obtained from Coleção de Porifera from the Universidade Federal de Pernambuco (UFPE) were subjected to the same procedure and analysis as described in Hardoim et al. (2021). The resulting sequences were submitted to the NCBI database under the accession numbers MW092803–MW092817.

### Phylogenetic Analyses of Marine Sponges

In total, 21 sequences (five from each sponge species, along with others obtained from Coleção de Porifera from UFPE) were used in the phylogenetic analyses. For phylogenetic inferences, cob gene sequences from marine demosponges were searched for using the megaBLAST and BLASTn algorithms of the National Centre for Biotechnology Information (NCBI; Altschul et al., 1990, 1997) and retrieved. In this case, the cob sequences were found from *Aplysina cauliformis* (Sperling et al., 2012) and *A. fulva* (Lavrov et al., 2008). Redundancy was removed and the final phylogeny encompassed 59 sequences. The alignment, selection of the best model, maximum likelihood, and Bayesian phylogenetic analyses were carried out as explained in detail in Hardoim et al. (2021). The best choice was given to the general-time reversible model (GTR; Rodríguez et al., 1990) with a discrete gamma distribution of among-site rate variation (Γ4) and a proportion of invariant sites (I).

### Illumina Sequencing

An aliquot of the purified genomic DNA was submitted to the Functional Genomics Center from the Luiz de Queiroz College of Agriculture (ESALQ-USP) for sequencing of the 16S and 18S rRNA genes and the internal transcribed spacer (ITS) of the ribosomal RNA gene operon following all appropriate quality controls. Briefly, the V4 region of the 16S rRNA gene, targeting the prokaryotic communities, was amplified with the primer pair 515F-806R (Apprill et al., 2015; Parada et al., 2016). For the ITS, the primer pair used was ITS1f-ITS2 (Gardes and Bruns, 1993; Smith and Peay, 2014), targeting the fungi communities. The V9 region of the 18S rRNA gene, aiming for the microbial eukaryotic communities, was amplified with the primer pair Euk1391f-EukBr (Amaral-Zettler et al., 2009; Stoeck et al., 2010). For a detailed description of the primer pair sequences, PCR reaction mixtures, and thermal cycles see the Supplementary Material. These amplicons were subjected to Illumina sequencing with the MiSeq platform.

### Illumina Data Analysis

Illumina sequences were processed in Mothur v. 1.44 (Schloss et al., 2009). For each dataset (16S and 18S rRNA genes and ITS), a pipeline was optimized and executed. For detailed description of the pipelines, see the Supplementary Material. Overall, the paired raw sequences were joined. The datasets were demultiplexed, reduced to non-identical sequences, and then sequences were aligned using the reference SILVA seed v. 138 database (Mothur-formatted); provided by Mothur (Quast et al., 2013) for 16S and 18S rRNA genes, and UNITE v. 8.2 dataset, Mothur-formatted for ITS (Abarenkov et al., 2020). Prior to chimera check, the sequences were pre-clustered. However, this step was not performed for the ITS. Then, chimeric sequences were detected with UCHIME (Edgar et al., 2011) and filtered out. Sequences were phylogenetically classified with the reference SILVA non-redundant v. 138 (Quast et al., 2013) database for 16S and 18S rRNA genes, and UNITE v. 8.2 for the ITS (Abarenkov et al., 2020). Undesirable sequences (e.g., mitochondria and chloroplast for the prokaryotic dataset, protist and metazoan for the fungi dataset, and Porifera and bacteria for the unicellular eukaryotes dataset, a detailed description is provided in the Supplementary Material) and singletons were removed from the dataset. Sequences were assigned to operational taxonomic units (OTUs) classified at 97% sequence similarity. The libraries were normalized. The representative sequences of each OTU were obtained. OTUs were further classified based on the SILVA non-redundant v. 138 database (Mothur-formatted; Quast et al., 2013) for 16S and 18S rRNA genes, and UNITE v. 8.2 for the ITS (Abarenkov et al., 2020). All datasets generated in this study were deposited as a Sequence Read Archive in the NCBI database with Bioproject ID: PRJNA673577 (SAMN16616778–SAMN16616802) for the 16S rRNA gene (SAMN16616803–SAMN16616827) for the 18S rRNA gene, and (SAMN16616828–SAMN16616852) for the ITS.

### Ecological Metrics and Statistical Analyses

Rarefaction curves for all datasets (16S and 18S rRNA genes and ITS) were performed as described in the Supplementary Material. Richness [observed OTUs, CHAO, and abundance-based coverage estimators (ACE)], diversity (Shannon – *H'* and inverse Simpson – *D*_2_), and evenness (Pielou’s evenness) indicators were calculated using the R package vegan v. 2.5-6 (Oksanen et al., 2019; R Core Team, 2020). ANOVA was performed for the alpha metrics using R package vegan 2.5-6 (Oksanen et al., 2019; R Core Team, 2020). For ANOVA, a value of *p* equal to or smaller than 0.001 was considered statistically significant. The multiple comparisons of means with Tukey contrasts were performed with the multcomp R package (Hothorn et al., 2016; R Core Team, 2020).

Phylogenetic signal analysis was undertaken to quantitatively measure the co-occurrence of species that share similar traits (Blomberg et al., 2003) with the diversity indices *H'* and *D*_2_ of prokaryotic, fungal, and unicellular eukaryotic communities. This analysis was computed with the host sponge phylogeny using the phylosignal function of the R package picante (Kembel et al., 2010; R Core Team, 2020). The phylogenetic signal compares whether the ecological signal from microorganisms is more closely related with the evolution of the host sponge than expected by chance.

Non-metric multidimensional scaling (nMDS) was used to summarize patterns of microbial community structure based on Bray–Curtis dissimilarity distance matrices using vegan package v. 2.5-6 (Oksanen et al., 2019; R Core Team, 2020). Permutational multivariate analysis of variance (PERMANOVA) performed with the Adonis function and analysis of similarity (ANOSIM) were used to test the significance of the differences across samples.

The datasets presented several OTUs associated with marine sponges that were unclassified at some level of taxonomy affiliation (from phylum to genus or to species in the case of the fungi dataset). To obtain more details, these OTUs were subjected to BLASTn (Altschul et al., 1990, 1997; Agarwala et al., 2016) using the SILVA non-redundant v. 138 database for the prokaryotic and unicellular eukaryotic datasets and the ITS RefSeq Targeted Loci (RTL) database from NCBI for the fungi dataset (Schoch et al., 2014). In this context, it is critical to note that standard NCBI annotation was used to collect host and isolation source information, when available. The scripts used are provided in the Supplementary Material.

The habitat generalists and specialists are key ecological groups of microbial communities. It is predicted that the microbial assembly of each group is controlled by different ecological process (Székely and Langenheder, 2014; Liao et al., 2016; Hou et al., 2019). Here, key habitat generalists and specialists from marine sponges and environmental samples were identified based on the Levins’ niche breadth (B) index with permutation algorithms (1,000 permutations) by using EcolUtils.^1^ This approach generated 1,000 stimulated OTU tables using the *quasiswap* permutation algorithm, and compared these results with the observed occurrences for the true microbial communities. An OTU was defined as a habitat generalist or as a habitat specialist when the observed occurrence exceeded the upper and the lower 95% CI, respectively (Wu et al., 2017). All OTUs observed within the 95% CI were considered as neutral taxa. This approach avoids the identification of habitat specialists due to rare occurrence.

To determine the shared and exclusive OTUs, a Venn diagram was constructed in R with the VennDiagram v. 1.6.20 R package (Chen and Boutros, 2011; R Core Team, 2020), whereas the taxonomic affiliations of the OTUs were discovered using the online tool jvenn^2^ (Bardou et al., 2014). In this study, the core OTUs were defined as OTUs present in all categories, but not necessarily in all replicates.

The Linear Discriminant Analysis (LDA) Effect Size (LEfSe; Segata et al., 2011) was carried out in the Galaxy web platform (Afgan et al., 2018) with default parameters to determine the phylogenetic lineages responsible for the differences detected in the categories (sponge species, seawater, and sediment).

### Estimating the Stochastic Ratio of Community Assembly

A modified stochasticity ratio (MST) analysis was undertaken to evaluate the relative importance of deterministic and stochastic processes to whole prokaryotic and eukaryotic communities’ assembly as well as in each partitioning (i.e., habitat generalists and habitat specialists). This metric estimates ecological stochasticity according to a null-model-based statistical framework as previously described (Guo et al., 2018). The value of MST index was developed with 50% as the boundary point to divide the deterministic-dominance (<50%) and stochastic-dominance (>50%) community assembly (Ning et al., 2019). The MST analysis was performed based on the taxonomic assignment by using Bray-Curtis distance and was implemented in the NST R package (Ning et al., 2019; R Core Team, 2020).

### Neutral Model Analysis

The potential contribution of neutral processes to microbial community assembly in each sample was assessed by the Sloan neutral model (Sloan et al., 2006; Burns et al., 2016). This model predicts the relationship between occurrence frequency of taxa and their relative abundance in the metacommunity (sum of all samples for each microhabitat). In general, more abundant taxa in the metacommunity are expected to be more widespread and be randomly sampled by an individual, whereas rare taxa are more likely to go extinct in different local communities because of ecological drift. In the model, the parameter *m* is the estimated immigration rate and the parameter *R*^2^ indicates the overall fit to the model (Sloan et al., 2006). The 95% CI of the model was calculated based on 1,000 bootstrap replicates. All sponge samples (*n* = 15) as well as environmental samples (*n* = 10) were used to predict the model in the R scripts as previously described (Burns et al., 2016).

## Results

### Sponge Identification

The analysis of 364 bp-long sequences of the cob gene obtained from all 15 specimens showed no intraspecific variations among our sequences of *A. caissara* and *A. fulva*, while a genetic distance (*p*-distance) between 0 and 0.55% was observed in the individuals of *T. ignis*. A *p*-distance of 0.15% was found among the three sponge species. The *p*-distance between our sequences and the ones obtained from Coleção de Porifera and from Genbank ranged between 0.54 and 0.84% for *A. caissara* and between 0 and 0.54% for *A. fulva*, respectively. Phylogenetic reconstructions based on maximum likelihood and Bayesian inferences showed that each sponge species formed a robust cluster (Supplementary Figure S1). The cob gene was shown to be efficient in separating *A. caissara* and *A. fulva* collected along the coast of São Sebastião.

### Prokaryotic Community Composition

A total of 3,751,766 V4-region 16S rRNA gene sequences were obtained. After denoising, quality filtering, removal of chimera, undesirables, and singletons, a total of 2,757,991 16S rRNA sequences were further rarefied to the same library depth of 72,105 sequences, resulting in 1,802,625 sequences (Supplementary Table S1). These were assigned to 40,469 OTUs at 97% sequence similarity.

By comparing the observed and expected OTU distribution, the habitat generalist and specialist groups in each sample were identified. Among them, 727 habitat generalist OTUs and 1,167 habitat specialist OTUs were identified across all samples that represented 1.8 and 2.9% of the total sequences, respectively. The total prokaryotic community associated with at least one sample from the three investigated sponge species was assigned to 18,580 OTUs (45.9% of the total dataset), of which 633 habitat generalist OTUs and 845 habitat specialist OTUs were represented by 1.6 and 2.1% of the total sequences, respectively.

#### Prokaryotic Alpha Ecological Metrics

The rarefaction curves demonstrated that none of the categories reached the plateau with the sequence depth used in this dataset, although *Aplysina* species, *T. ignis*, and seawater were closer to the plateau than sediment (Supplementary Figure S2). The results among the investigated richness indices were very similar. The highest observed OTUs, CHAO, and ACE richness indices were detected in sediment, followed by seawater, *A. caissara*, *A. fulva*, and *T. ignis* (Table 1). Mean pairwise corrections for each of these indices revealed significant differences (*p* < 0.001) between sediment and all other categories, as well as between seawater and *T. ignis* (Supplementary Table S2A). Similar to the investigated richness indices, the results from *H'* and *D*_2_ diversity and Pielou’s evenness indices ranged from the largest values encountered in sediment followed by *Aplysina* species, seawater, and down to the lowest values found in *T. ignis* (Table 1). For *H'* and Pielou’s evenness indices, all the combinations between two categories were significantly different (*p* < 0.05; Supplementary Table S2A).

**Table 1:** Values for richness, diversity, and evenness indices.

Dataset	Ecological metrics	Ac	Af	Ti	SW	SD
Prokaryotes	Observed OTUs	2801.4 ± 46.12	2723.2 ± 173.30	2282.6 ± 211.51	3253.6 ± 45.98	9551.2 ± 138.09
	CHAO	6124.05 ± 146.35	5919.23 ± 327.20	3746.83 ± 287.07	6477.90 ± 108.91	13737.95 ± 210.93
	ACE	6794.88 ± 182.13	6655.91 ± 375.36	3903.75 ± 192.89	7387.22 ± 132.64	14894.14 ± 240.04
	Shannon (*H'*)	4.75 ± 0.068	5.13 ± 0.080	2.90 ± 0.12	3.94 ± 0.023	7.45 ± 0.04
	Inverse Simpson (*D*_2_)	42.20 ± 7.24	61.55 ± 11.30	4.33 ± 0.32	9.96 ± 0.21	241.94 ± 19.70
	Pielou’s evenness	0.60 ± 0.0078	0.65 ± 0.015	0.37 ± 0.014	0.50 ± 0.002	0.81 ± 0.003
Fungi	Observed OTUs	42.2 ± 7.2	37 ± 6.98	22.6 ± 5.14	126.6 ± 9.46	37.6 ± 1.6
	CHAO	60.50 ± 9.39	69.53 ± 11.28	40.6 ± 11.78	201.73 ± 12.32	54.78 ± 6.08
	ACE	61.45 ± 10.52	64.53 ± 8.15	47.33 ± 10.99	201.02 ± 12.39	55.47 ± 4.59
	Shannon (*H'*)	2.89 ± 0.26	2.55 ± 0.25	1.40 ± 0.11	3.95 ± 0.37	2.17 ± 0.16
	Inverse Simpson (*D*_2_)	12.81 ± 2.26	8.61 ± 2.01	2.92 ± 0.52	33.30 ± 8.18	4.97 ± 0.83
	Pielou’s evenness	0.78 ± 0.038	0.72 ± 0.035	0.47 ± 0.05	0.81 ± 0.07	0.59 ± 0.04
Unicellular eukaryotes	Observed OTUs	27 ± 3.86	28.2 ± 4.46	34 ± 4.87	32 ± 1.30	63.4 ± 1.75
	CHAO	57.56 ± 10.31	41.15 ± 10.74	94.37 ± 17.03	136.26 ± 25.25	181.13 ± 10.48
	ACE	79.69 ± 15.91	45.60 ± 12.08	100.65 ± 18.65	132.85 ± 17.04	212.58 ± 23.05
	Shannon (*H'*)	1.83 ± 0.16	2.51 ± 0.31	2.57 ± 0.27	2.41 ± 0.12	3.73 ± 0.05
	Inverse Simpson (*D*_2_)	2.87 ± 0.18	9.44 ± 2.96	7.56 ± 1.32	5.75 ± 0.54	25.42 ± 2.36
	Pielou’s evenness	0.56 ± 0.02	0.76 ± 0.07	0.73 ± 0.04	0.70 ± 0.02	0.90 ± 0.008

Ac, *Aplysina caissara*; Af, *Aplysina fulva*; Ti, *Tedania ignis*; SW, seawater; and SD, sediment.

We found a significant phylogenetic signal when we compared the evolutionary inference of sponge species with diversity indices of *H'* (*K* = 1.9e^−4^, *p* < 0.001) and *D*_2_ (*K* = 1.5e^−5^, *p* < 0.05) for the whole prokaryotic community. This suggested a convergent pattern of evolution between sponge species and the diversity of their associated prokaryotic community, with samples of *A. fulva* and *A. caissara* displaying significantly larger values of *H'* and *D*_2_ than *T. ignis* replicates (Table 1).

#### Prokaryotic Beta Ecological Metrics

**Figure 1:**
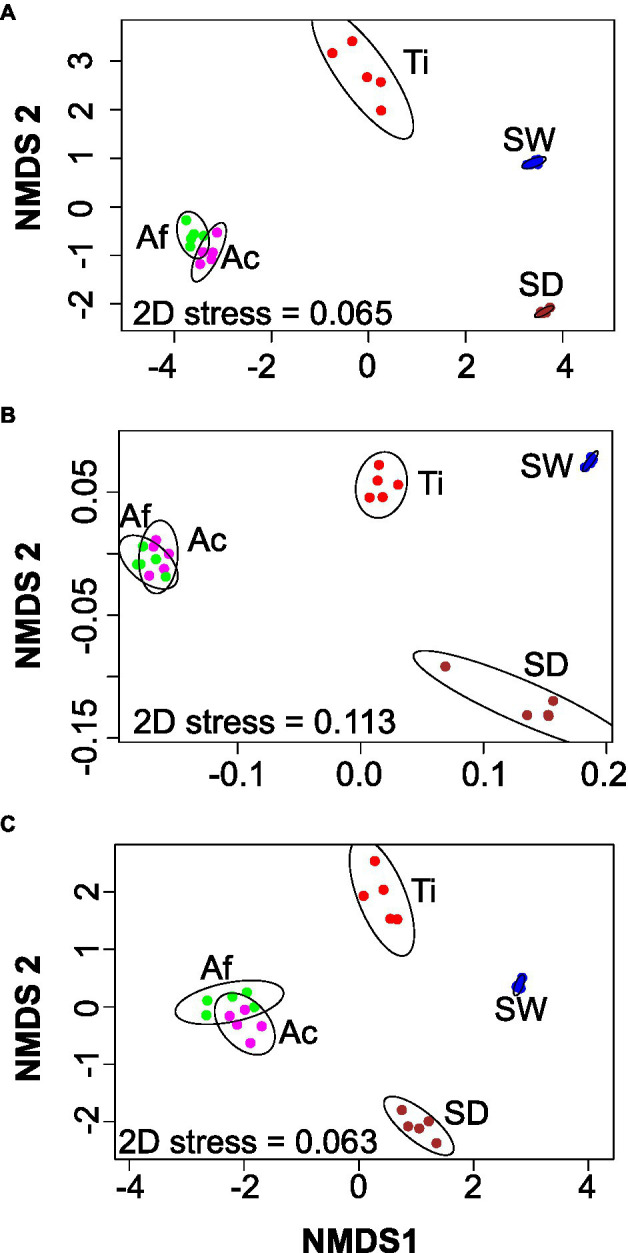
Non-metric multidimensional scaling (nMDS) based on Bray–Curtis distances for the whole **(A)**, habitat generalist **(B)**, and habitat specialist **(C)** prokaryotic community. Ac, *Aplysina caissara*; Af, *Aplysina fulva*; Ti, *Tedania ignis*; SW, seawater; and SD, sediment.

Non-metric dimension scaling ordination analysis demonstrated four main groups: (i) *Aplysina* species, (ii) sediment, (iii) seawater, and (iv) *T. ignis*, either for the whole community or for the habitat generalist-specialist groups (Figure 1). Replicates from *T. ignis* were by far the most dissimilar for the whole community (Figure 1A), while sediment samples were also largely dissimilar for habitat generalists (Figure 1B). Replicates from each treatment were very similar for habitat specialists (Figure 1C). PERMANOVA and ANOSIM further confirmed that the compositions of the whole communities and the habitat generalists-specialists were significantly different (*p* < 0.001) with high goodness-of-fit values (*R*^2^ > 0.88; Supplementary Table S3).

#### Community Composition at Phyla and Class Levels

**Figure 2:**
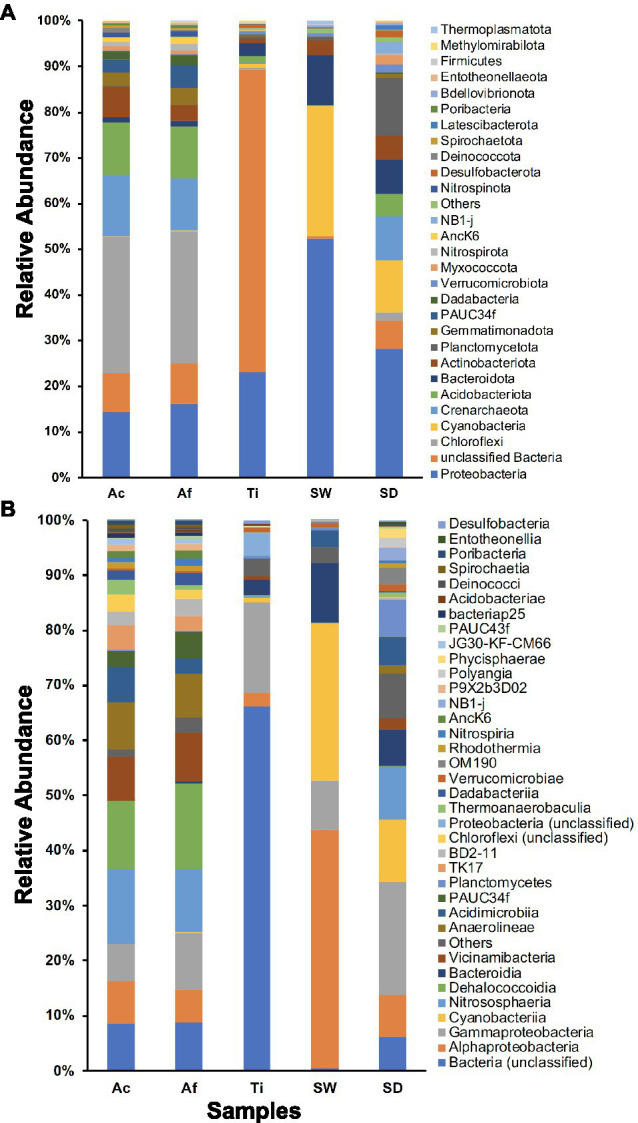
Prokaryotic community composition for Ac (*Aplysina caissara*), Af (*Aplysina fulva*), Ti (*Tedania ignis*), SW (seawater), and SD (sediment) is shown for 28 prokaryotic phyla **(A)** and for 36 classes with relative abundance >0.2% **(B)**.

In total, 63 prokaryotic phyla were retrieved in the whole community. Sediment samples encompassed the most diverse community composition with 59 phyla, followed by seawater, *T. ignis*, *A. fulva*, and *A. caissara* with 58, 44, 42, and 37 phyla, respectively. The three sponge species together comprised 49 phyla (Supplementary Table S4A). The prokaryotic communities associated with *A. caissara* and *A. fulva* were very similar and were dominated by Chloroflexi, Proteobacteria, Crenarchaeota, and Acidobacteriota (Figure 2A; Supplementary Table S4A). Unclassified bacteria and Proteobacteria dominated the prokaryotic community composition associated with *T. ignis*. Proteobacteria, Cyanobacteria, and Bacteroidota dominated seawater samples. Proteobacteria, Planctomycetota, and Cyanobacteria were the most abundant prokaryotic phyla detected in the sediment samples. Similar patterns of community composition were also observed for habitat generalist and specialist groups (Supplementary Figure S3). For habitat generalists, *Aplysina* species were dominated by Chloroflexi as was observed for the whole dataset (Supplementary Figure S3A), whereas for the habitat specialists, Proteobacteria was the most abundant phylum associated with these hosts (Supplementary Figure S3C). The community composition of habitat generalists in *T. ignis* was largely represented by unclassified bacteria and Proteobacteria (Supplementary Figure S3A).

A total of 163 prokaryotic classes were also recovered in the whole community. A similar pattern as observed for phylum level was detected here, with decreasing diversity from sediment over seawater, *T. ignis*, and *Aplysina* species (Figure 2B; Supplementary Table S4B). For *A. caissara*, Nitrososphaeria, Dehalococcoidia, unclassified bacteria, and Anaerolineae dominated the community composition. Dehalococcoidia, Nitrososphaeria, Gammaproteobacteria, and Vicinamibacteria were the most abundant classes associated with *A. fulva*. The most dominant classes associated with *T. ignis* were unclassified bacteria and Gammaproteobacteria. Alphaproteobacteria, Cyanobacteria, Bacteroidia, and Gammaproteobacteria dominated the community composition obtained from seawater samples. In sediment samples the most dominant prokaryotic classes were Gammaproteobacteria, Cyanobacteria, and Nitrososphaeria. For the habitat generalist community, the most dominant class associated with *T. ignis* was unclassified bacteria, while Dehalococcoidia was the most abundant class associated with *A. fulva* (Supplementary Figure S3B). The class level of habitat specialist community was largely heterogeneous for all investigated sponge species with the dominant class assigned to Gammaproteobacteria for *A. fulva* and *T. ignis*, and Nitrososphaeria for *A. caissara* (Supplementary Figure S3D).

The unclassified lineages associated with *A. caissara*, *A. fulva*, and *T. ignis* were further investigated at the OTU level. The analysis revealed that 9,730 OTUs (52.37% of assigned sponge-associated community) were unclassified at some level of the taxonomy affiliation. Further analyses showed that 77.1% of these OTUs presented similarity below 97% and among them, 41.1% have been associated with other sponge species (Supplementary Tables S5A,B).

#### Assemblage of Prokaryotic Community

**Figure 3:**
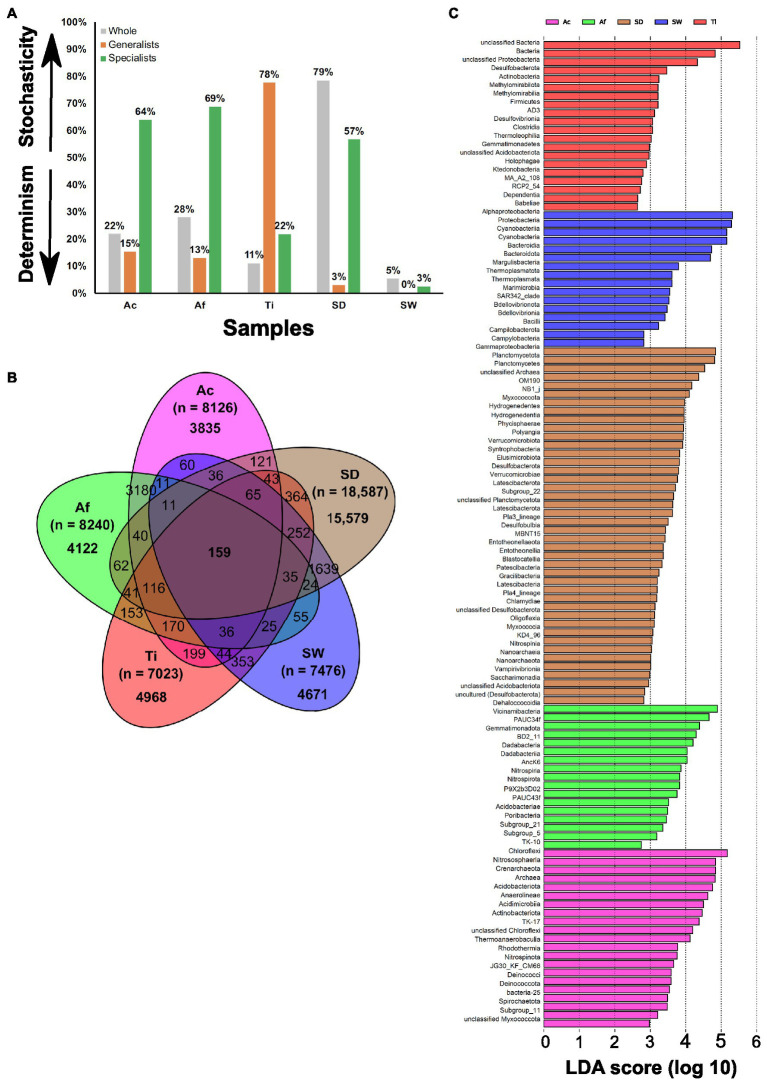
The modified stochasticity ratio (MST) of prokaryotic community under different groups. Ac (*Aplysina caissara*), Af (*Aplysina fulva*), Ti (*Tedania ignis*), SW (seawater), and SD (sediment) **(A)**, venn diagram with all OTUs detected in Ac (magenta), Af (green), Ti (red), SW (blue), and SD (brown) **(B)**, and taxonomic representation of statistically and biologically consistent differences across Ac, Af, Ti, SW, and SD **(C)**.

The MST estimated with Bray–Curtis dissimilarity metric revealed that the whole prokaryotic community in all the sponge species was more strongly driven by deterministic assembly processes (MST < 50%), in which samples from *T. ignis* exhibited the lowest stochasticity ratio (Figure 3A; Supplementary Table S6A). These samples did not show a good fit to the Sloan neutral community model prediction, due to a better fit to the randomly sampling of the source metacommunity (*R*^2^.pois) than the fit observed within the sample (*R*^2^) or by presenting a negative *R*^2^-value (Supplementary Table S6B). This suggests the predominance of deterministic processes in shaping the prokaryotic assemblage in the sponge species. The habitat generalist community associated with *Aplysina* species and environmental samples were demonstrated to be dominated by deterministic assemblage processes, whereas habitat generalists associated with *T. ignis* revealed the highest stochasticity ratio (77.6%) compared to all other samples (Figure 3A). None of the habitat generalist communities showed a good fit to the Sloan neutral community model prediction (Supplementary Table S6B). The habitat specialist community associated with *Aplysina* species and sediment samples were demonstrated to be assembled by stochasticity processes, while habitat specialists associated with *T. ignis* and seawater samples were revealed to be dominated by deterministic assembly processes (Figure 3A). The Sloan neutral model was well fitted to the habitat specialists associated with *A. caissara* and sediment samples, and showed lower *R*^2^ values with *A. fulva* and *T. ignis* samples (Supplementary Figure S4; Supplementary Table S6B). The estimated migration rate (*m*) of habitat specialists was larger for sediment samples (*m* = 0.23) than for *A. caissara* (*m* = 0.1), which suggested a higher dispersion limitation within sponge habitat specialists than sediment samples.

#### Specificities and Commonalities: Shared and Exclusive OTUs

The Venn diagram demonstrated that the vast majority of OTUs (33,175) was specific of each category and very few (159 OTUs) were shared among all of them (Figure 3B). Overall, unclassified bacteria represented the most abundant OTUs exclusively associated with each sponge species (Supplementary Tables S7A–D). Among the identifiable classes the most abundant in *A. caissara* were Alphaproteobacteria, Dehalococcoidia, and Gammaproteobacteria. Around 47% of its prokaryotic community was specific to this host species (Supplementary Table S7A). In *A. fulva*, 50% of its prokaryotic community was specific and among the recognized classes the most abundant were Gammaproteobacteria, Dehalococcoidia, and Vicinamibacteria (Supplementary Table S7B). The *Aplysina* species shared 25% of the prokaryotic community (Supplementary Table S7C). The most dominant identifiable classes were Dehalococcoidia, Anaerolineae, and Gammaproteobacteria (Supplementary Table S7C). In *T. ignis*, 70.7% of its prokaryotic community was host-specific and the most abundant recognized classes were Gammaproteobacteria and Alphaproteobacteria (Supplementary Table S7D). The sponge core encompassed 170 OTUs and was dominated by Dehalococcoidia, unclassified bacteria, Alphaproteobacteria, and Vicinamibacteria (Supplementary Table S7E).

For the environmental samples, seawater had 62.5% of its prokaryotic community specific to it and the most dominant classes were Alphaproteobacteria, Cyanobacteria, Gammaproteobacteria, and Bacteroidia (Supplementary Table S7F). In sediment, 84% of its prokaryotic community was specific to this category and the most dominant classes were Gammaproteobacteria, unclassified bacteria, Planctomycetes, and Bacteroidia (Supplementary Table S7G). The most abundant classes in the core were Gammaproteobacteria, Alphaproteobacteria, unclassified bacteria, and Nitrososphaeria (Supplementary Table S7H).

The habitat generalist group was composed of 10 dominant phyla and the tree demonstrated that only a few phylogenetic-related OTUs were common to more than one category (Supplementary Figure S5). The habitat specialist group encompassed 14 abundant phyla and in opposition to the habitat generalist, the habitat specialist tree showed that several phylogenetic-related OTUs were detected in more than one category (Supplementary Figure S6).

#### LEfSe Analysis

From all taxonomic affiliations classified until class level, 116 could explain the variability detected in the whole prokaryotic communities associated with sponge species and in the environmental samples. Sediment had 42 lineages, followed by *A. caissara*, *A. fulva*, *T. ignis*, and seawater with 21, 20, 17, and 16, respectively (Figure 3C; Supplementary Table S8A). Each sponge species and environmental sample enriched its own set of lineages that did not overlap (Supplementary Table S8A). Here, an LDA score above 4.5 is presented; for the entire list of enriched lineages see Supplementary Table S8A. For *A. caissara*, the lineages were affiliated to Chloroflexi, Nitrososphaeria, Crenarchaeota, Archaea, Acidobacteriota, and Anaerolineae. For *A. fulva*, the lineages were affiliated to Dehalococcoidia and Vicinamibacteria. For *T. ignis*, the lineages were affiliated to unclassified bacteria and bacteria. For seawater, the lineages were Alphaproteobacteria, Proteobacteria, Cyanobacteria, Bacteroidia, and Bacteroidota. For sediment, the lineages were Gammaproteobacteria, Planctomycetota, and Planctomycetes. Only few lineages (*n* = 8) were significantly enriched among the habitat generalist group associated with sponge species (Supplementary Table S8B): two members from classes Anaerolineae and Alphaproteobacteria in *A. caissara*, members of Dadabacteriia and Vicinimabacteria in *A. fulva*, and a member from Proteobacteria in *T. ignis* (Supplementary Table S8B). Similar to the whole prokaryotic community, the habitat specialist group associated with sponge species was also enriched with a large number (*n* = 39) of lineages (Supplementary Table S8C) that resembled the results from the Venn diagram analysis. The members of habitat specialist group enriched in *A. caissara* (*n* = 17) were largely heterogeneous, while members of Vicinamibacteria and PAUC34f were among the most enriched classes in *A. fulva* (*n* = 19) and a member of unclassified bacteria was enriched in *T. ignis* (*n* = 3).

### Eukaryotic Community Composition

#### Fungal Dataset

A total of 1,895,188 ITS region sequences were acquired. After denoising, quality filtering, and removal of chimera, undesirables, and singletons, 929,307 ITS region sequences were further rarefied to 470 sequences in each library with a total of 11,750 sequences (Supplementary Table S9). We observed an extremely low sequence recovered after quality control of *T. ignis* samples. This sponge species ranged from 480 to 7,404 sequences, in contrast with all other categories (*A. caissara*, *A. fulva*, seawater, and sediment that ranged from 47,001–54,378, 43,584–56,674, 29,707–40,688, and 41,047–53,966 sequences, respectively). Therefore, two analyses were performed: (i) with all 25 replicates with a depth of 470 sequences and (ii) without *T. ignis* samples with a depth of 28,812 sequences. Results from the former are presented below, whereas results from the latter are presented in the Supplementary Material. The rarefied 470 sequences were assigned to 958 OTUs at 97% sequence similarity.

We also compared the observed and expected OTU distribution to detect habitat generalist and specialist groups in the fungal community. Among the rarefied 958 OTUs in the whole fungal community, 26 habitat generalist OTUs and eight habitat specialist OTUs were identified across all samples that represented 2.7 and 0.8% of the total sequences, respectively. The total fungal community associated with at least one sample from the three investigated sponge species was assigned to 397 OTUs (41.4% of the total dataset), of which nine habitat generalist OTUs (2.3% of assigned sponge OTUs) and only one specialist OTU from *T. ignis* were represented. Because of the low representativeness of habitat generalist and specialist groups across sponge species, we have only shown their community composition at the lowest taxonomic level possible (Supplementary Figures S7A,B, respectively), without further community analyses.

#### Fungal Alpha Ecological Metrics

The rarefaction curves showed that seawater did not reach the plateau, whereas the other categories were approaching the plateau (Supplementary Figure S8A). The investigated richness, diversity, and evenness indices were highest in seawater, followed by *Aplysina* species, sediment, and *T. ignis* (Table 1). Mean pairwise comparisons revealed a significant (*p* < 0.001) difference between seawater and all other categories for richness indices, between seawater and *T. ignis* for diversity indices, and a significant difference (*p* < 0.01) between *A. caissara* and *T. ignis*, and *T. ignis* and seawater for Pielou’s evenness (Supplementary Table S2B).

As observed in the prokaryotic community, a significant phylogenetic signal was also observed for the whole fungal community when comparing sponge species evolutionary inference with diversity indices of *H'* (*K* = 1.9e^−5^, *p* < 0.05) and *D*_2_ (*K* = 1.7e^−5^, *p* < 0.05). Samples of *Aplysina* species displayed significantly larger values of *H'* and *D*_2_ than *T. ignis* replicates (Table 1).

#### Fungal Beta Ecological Metrics

**Figure 4:**
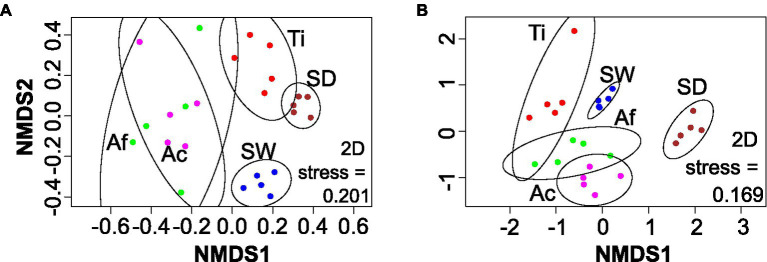
nMDS based on Bray–Curtis distances for the fungi **(A)** and unicellular eukaryotic **(B)** datasets. Ac, *Aplysina caissara*; Af, *Aplysina fulva*; Ti, *Tedania ignis*; SW, seawater; and SD, sediment.

The nMDS of fungal community also revealed four main groups: (i) *Aplysina* species, (ii) *T. ignis*, (iii) sediment, and (iv) seawater (Figure 4A). Sponge replicates presented a higher dissimilarity distribution, especially *Aplysina* species, than environmental replicates. Replicates from *Aplysina* species were grouped on one side of the ordination apart from *T. ignis* and environmental samples. PERMANOVA and ANOSIM confirmed that the differences observed among sponge species, seawater, and sediment were significant (*p* < 0.001; Supplementary Table S3).

#### Community Composition at High Taxonomic Ranks

**Figure 5:**
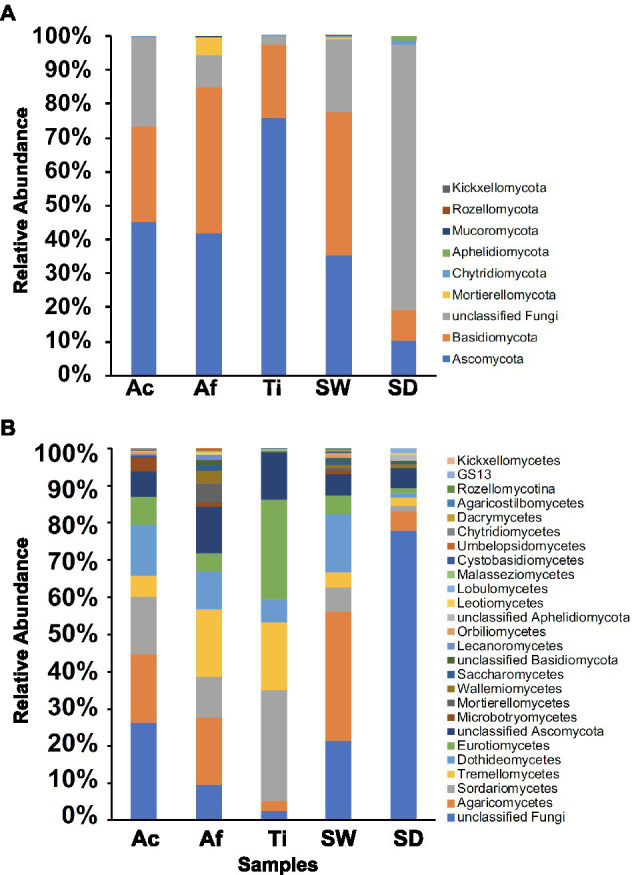
Fungal community composition for Ac (*Aplysina caissara*), Af (*Aplysina fulva*), Ti (*Tedania ignis*), SW (seawater), and SD (sediment) are shown for nine phyla **(A)** and for 26 classes **(B)**.

Overall, 10 fungal phyla were retrieved. Seawater encompassed seven fungal phyla followed by *A. fulva* and sediment with five each, whereas *A. caissara* and *T. ignis* were represented each by four phyla (Figure 5A; Supplementary Table S10A). Together, the three sponge species comprised seven phyla. For *A. caissara*, Ascomycota, Basidiomycota, and unclassified fungi were the most dominant phyla. Basidiomycota and Ascomycota were the most abundant phyla recovered from *A. fulva*. For *T. ignis*, the most abundant phylum was Ascomycota. For seawater, Basidiomycota, Ascomycota, and unclassified fungi were the dominant phyla. For sediment, unclassified fungi was the dominant phylum.

In total, 26 fungal classes were detected among categories. The most diverse community composition was detected in seawater with 22 classes, followed by *A. fulva*, *A. caissara*, sediment, and *T. ignis* with 18, 6, 6, and 14 classes, respectively (Figure 5B; Supplementary Table S10B). The three sponge species together encompassed 21 classes. The classes unclassified fungi, Agaricomycetes, Sordariomycetes, and Dothideomycetes were the most abundantly associated with *A. caissara*. For *A. fulva*, the most dominant classes were Agaricomycetes, Tremellomycetes, unclassified Ascomycota, and Sordariomycetes. In *T. ignis*, the following classes were dominant: Sordariomycetes, Eurotiomycetes, Tremellomycetes, and unclassified Ascomycota. For seawater the most dominant classes were Agaricomycetes, unclassified fungi, and Dothideomycetes. For sediment, unclassified fungi was the dominant class.

The unclassified lineages associated with the three sponge species were further analyzed at the OTU level. It showed that 231 OTUs (58% of the assigned sponge-associated community) were unclassified at some level of the taxonomy affiliation. Further analyses revealed that 68.8% of the OTUs presented similarity below 97% (Supplementary Tables S11A,B).

#### Assemblage of Fungal Community

**Figure 6:**
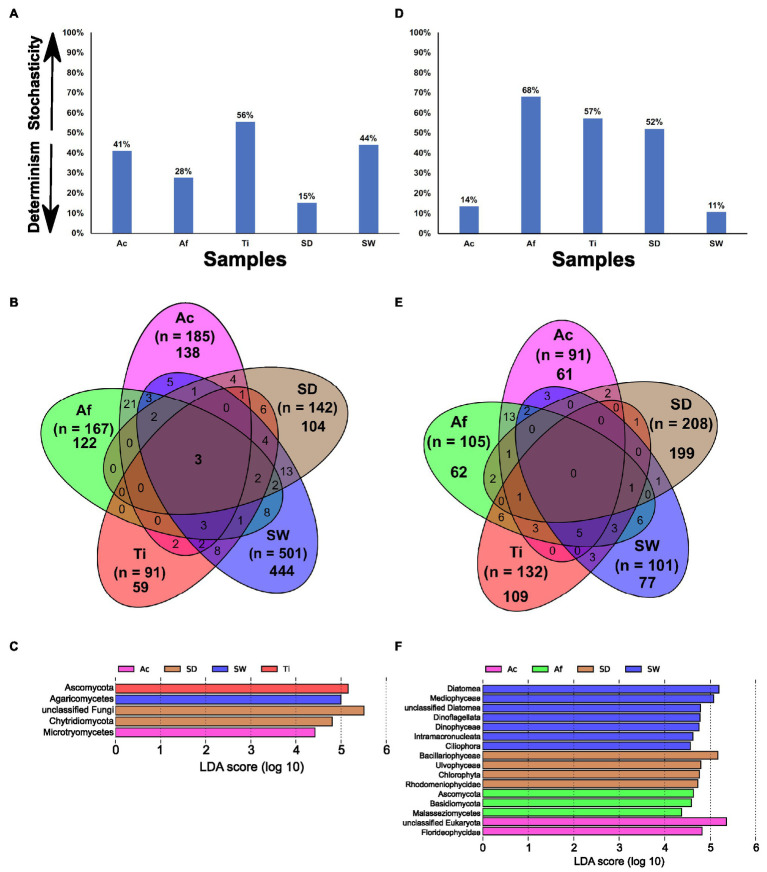
The MST, Venn diagram with all OTUs detected in Ac (*Aplysina caissara*, magenta), Af (*Aplysina fulva*, green), Ti (*Tedania ignis*, red), SW (seawater, blue), and SD (sediment, brown), and taxonomic representation of statistically and biologically consistent differences across Ac, Af, Ti, SW, and SD are presented for fungal **(A–C)** and unicellular eukaryotic **(D–F)** datasets, respectively.

The MST analysis performed with the fungal community revealed that all samples were driven by deterministic assembly processes (MST < 50%), except *T. ignis* (Figure 6A). Among sponge species, *A. fulva* exhibited the lowest stochasticity ratio (28%). Only replicates from *T. ignis* fitted to the Sloan neutral community model prediction (Supplementary Table S6B), supporting the predominance of deterministic processes shaping the fungal assemblage in the *Aplysina* sponge species and the stochasticity-dominance in the *T. ignis* samples.

#### Specificities and Commonalities: Shared and Exclusive OTUs

The Venn diagram showed that from a total of 958 OTUs, 867 were exclusively observed in the categories, whereas only three were common to all categories (Figure 6B). For *A. caissara*, 75.6% of its fungal community was specific of this host sponge and the most abundant classes were affiliated to unclassified fungi, followed by Dothideomycetes, and Eurotiomycetes (Supplementary Table S12A). For *A. fulva*, 73.0% of its fungal community was host-specific, and the most dominant classes were affiliated to Agaricomycetes, unclassified Ascomycota, and unclassified fungi (Supplementary Table S12B). For *T. ignis*, 64.8% of its fungal community was specific to it. The classes Sordariomycetes and Tremellomycetes were the most abundantly found in this sponge host (Supplementary Table S12C). None of the assigned OTUs were exclusively encountered in all sponge species. For seawater, 88.6% of its fungal community was specific of this category and the most abundant classes were affiliated to Agaricomycetes, unclassified fungi, and Dothideomycetes (Supplementary Table S12D). For sediment, 73.2% of its fungal community was registered in this category. The class unclassified fungi was found as the most abundant in sediment (Supplementary Table S12E). The core encompassed only three OTUs (0.5%) that were affiliated to the classes Dothideomycetes and Agaricomycetes (Supplementary Table S12F).

#### LEfSe Analysis

From all taxonomic affiliations classified until class level, only five could explain the variability identified in the whole fungal community associated with sponge species and detected in environmental samples. The lineage for *A. caissara* and *T. ignis* was represented by Microbotryomycetes and Ascomycota, respectively (Figure 6C; Supplementary Table S13). Seawater was represented by Agaricomycetes, and sediment by unclassified fungi and Chytridiomycota.

### Unicellular Eukaryotic Dataset

In total, 3,648,134 V9-region 18S rRNA gene sequences were obtained. After quality filtering, removal of chimera, undesirable sequences, including eukaryotic sequences other than the unicellular ones, and singletons, 885,767 sequences were rarefied to the same library depth of 119 sequences, resulting in 2,975 sequences that were assigned to 561 OTUs at 97% sequence similarity (Supplementary Table S14). In this dataset, *A. fulva* presented the lowest number of sequences (114–477) after quality control, compared to all other categories (*A. caissara*, *T. ignis*, seawater, and sediment ranged from 426–1,797, 1,094–4,099, 86,990–105,157, and 73,729–82,385, respectively). Based on this, two analyses were carried out: (i) with all five replicates from each category with a depth of 119 sequences and (ii) without *A. fulva* and two replicates from *A. caissara* with a depth of 700 sequences. Results from the former are presented below, whereas results from the latter are in the Supplementary Material.

By comparing the observed and expected OTU distribution we were able to detect habitat generalist and specialist groups in the unicellular eukaryotic community. A total of six OTUs (1.1% of the total sequences) were assigned as habitat generalists and all of them were exclusively detected in sponge species (Supplementary Figure S7C). Another 13 OTUs were assigned as habitat specialists across all samples and represented 2.3% of the total sequences (Supplementary Figure S7D). When we considered only those associated with at least one sample from the three sponge species, a total of 285 OTUs (50.8% of the total dataset) were encountered, of which six were assigned as habitat generalist OTUs and seven as habitat specialist OTUs that represented 2.1 and 2.5% of assigned sponge OTUs, respectively. Once again, because of the low representativeness of habitat generalist and specialist groups across sponge species, we have only shown their community composition at the lowest taxonomic level possible (Supplementary Figures S7C,D, respectively), without further community analyses.

#### Unicellular Eukaryotic Alpha Ecological Metrics

The rarefaction curves demonstrated that none of the categories reached the plateau (Supplementary Figure S8B). Once again, the results among the investigated richness indices were congruent. The highest observed OTUs, CHAO, and ACE richness indices were detected in sediment, then for observed OTUs it was followed by *T. ignis*, seawater, *A. fulva*, and *A. caissara*, and for CHAO and ACE it was followed by seawater, *T. ignis*, *A. caissara*, and *A. fulva* (Table 1). The results from *H'* and *D*_2_ diversity indices ranged from the largest values encountered in sediment followed by *T. ignis*, *A. fulva*, seawater, and *A. caissara*. Mean pairwise comparisons revealed significant dissimilarities (*p* < 0.01) for richness and diversity indices between sediment and all other categories (Supplementary Table S2C). The Pielou’s evenness index was highest for sediment, followed by *A. fulva*, *T. ignis*, seawater, and *A. caissara*. Mean pairwise comparisons exhibited significant difference between sediment and *A. caissara* (*p* < 0.001), and between *A. caissara* and *A. fulva*, *T. ignis*, and *A. caissara*, as well as between sediment and seawater (*p* < 0.05).

For the unicellular eukaryotic community, no significant (*p* > 0.05) phylogenetic signal was observed when we compared host evolutionary inference with diversity indices of *H'* and *D*_2_, which suggested that a random distribution of the data had a higher value for the goodness-of-fit than the observed sponge species relatedness.

#### Unicellular Eukaryotic Beta Ecological Metrics

The nMDS revealed five groups, one for each category (Figure 4B). Replicates from seawater formed a concise cluster, whereas the other categories presented a more dissimilar pattern, with samples from the same category apart from each other, especially for *A. fulva* and *T. ignis*. Nevertheless, PERMANOVA and ANOSIM confirmed that the differences observed among sponge species, seawater, and sediment were significant (*p* = 0.001; Supplementary Table S3).

#### Community Composition at High Taxonomic Ranks

**Figure 7:**
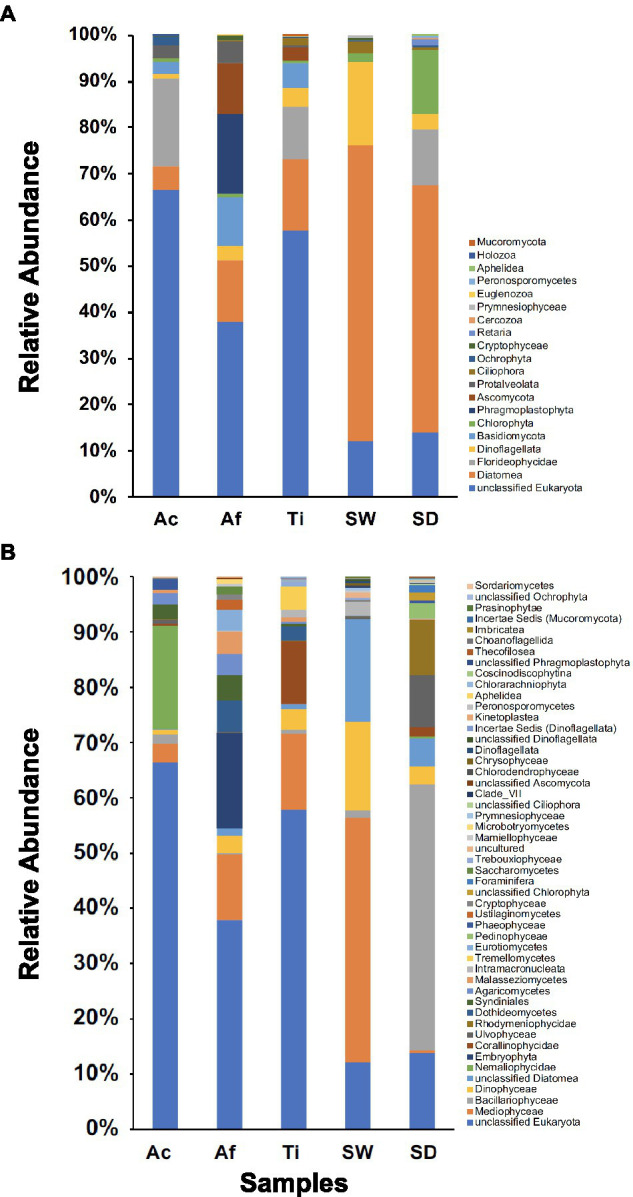
Unicellular eukaryotic community composition for Ac (*Aplysina caissara*), Af (*Aplysina fulva*), Ti (*Tedania ignis*), SW (seawater), and SD (sediment) are shown for 20 phyla **(A)** and for 50 classes **(B)**.

In total, 20 microbial eukaryotic phyla were recovered. Sediment encompassed the most diverse community composition with 13 phyla, followed by *T. ignis* with 12 phyla, whereas seawater, *A. caissara*, and *A. fulva* each comprised 11 phyla. Together, the three sponge species comprised 15 phyla (Figure 7A; Supplementary Table S15A). Unclassified Eukaryota was the most abundant phylum associated with the three sponge species. For *A. caissara*, the dominant identifiable phylum was Florideophycidae. For *A. fulva*, Phragmoplastophyta and Diatomea were also abundant. For *T. ignis*, Diatomea and Florideophycidae were dominant as well. Diatomea and Dinoflagellata were the most dominant phyla detected in seawater. For sediment, the most dominant phyla were Diatomea, Chlorophyta, and unclassified Eukaryota.

In total, 50 classes were observed among samples. The most diverse community composition was detected in sediment with 23 classes, followed by seawater with 22, *A. fulva* and *T. ignis* with 20 each, and *A. caissara* with 16 (Figure 7B; Supplementary Table S15B). *A. caissara*, *A. fulva*, and *T. ignis* together encompassed 32 classes. Unclassified Eukaryota was the most abundant class associated with the three sponge species. For *A. caissara*, Nemaliophycidae was the second most abundant class. Embryophyta and Mediophyceae also dominated the classes associated with *A. fulva*. For *T. ignis*, the classes Mediophyceae and Corallinophycidae were also abundant. For seawater, the most dominant classes were Mediophyceae and unclassified Diatomea. For sediment, Bacillariophyceae and unclassified Eukaryota were the most dominant classes.

To further explore the unclassified lineages associated with *A. caissara*, *A. fulva*, and *T. ignis*, analyses at the OTU level were performed. Overall, 193 OTUs (67.7% of the assigned sponge-associated community) were unclassified at some level of the taxonomy affiliation. Further analyses showed that 53% of the unclassified OTUs showed similarity below 97 and 19% of the unclassified OTUs did not fulfill our BLASTn requirements resulting in no matches against the reference database (Supplementary Tables S16A,B).

#### Assemblage of Unicellular Eukaryotic Community

The MST analysis performed with unicellular eukaryotic community revealed that *A. fulva*, *T. ignis*, and sediment samples were driven by stochasticity assembly processes (MST > 50%; Figure 6D). Among sponge species, samples from *A. caissara* exhibited the lowest stochasticity ratio (14%), supporting the predominance of deterministic assembly processes. None of the unicellular eukaryotic communities in the samples fitted well to the Sloan neutral model prediction (Supplementary Table S6B).

#### Specificities and Commonalities: Shared and Exclusive OTUs

The Venn diagram revealed that 508 OTUs from a total of 561 were specific to each category and none were common to all categories (Figure 6E). Overall, the unclassified Eukaryota was the most abundant lineage associated with *A. caissara*, *A. fulva*, and *T. ignis* (Supplementary Tables S17A–C). For *A. caissara*, 67% of its microbial eukaryotic was host-specific and the second most abundant OTU was affiliated to Nemaliophycidae (Supplementary Table S17A). For *A. fulva*, 59.1% of the unicellular eukaryotes were specific to this host species, and among the recognized lineages, Embryophyta also dominated (Supplementary Table S17B). For *T. ignis*, 82.6% of its microbial eukaryotes were species-specific, and among the identifiable classes, Corallinophycidae was abundant as well (Supplementary Table S17C). Only three OTUs were commonly detected across all sponge species (Supplementary Table S17D). For seawater, 72.2% of the unicellular eukaryotes were specific to it, and unclassified Eukaryota and Dinophyceae were the most dominant classes (Supplementary Table S17E). For sediment, 95.7% of its unicellular eukaryotes were specifically detected in this category and the most abundant classes were Bacillariophyceae and unclassified Eukaryota (Supplementary Table S17F).

#### LEfSe Analysis

Only 16 lineages from all taxonomic affiliations classified could explain the variability observed in the unicellular eukaryotic community associated with sponge species and detected in environmental samples. Seawater had seven lineages, followed by sediment with four, *A. fulva* with three, and *A. caissara* with two (Figure 6F; Supplementary Table S18). The lineages are presented in order of the highest to lowest LDA score values, with unclassified Eukaryota and Florideophycidae affiliated to *A. caissara*. For *A. fulva*, lineages were affiliated to Ascomycota, Basidiomycota, and Malasseziomycetes. The lineages Diatomea, Mediophyceae, unclassified Diatomea, Dinoflagellata, Dinophyceae, Intramacronucleata, and Ciliophora were enriched in seawater samples, whereas for sediment, lineages were affiliated to Bacillariophyceae, Ulvophyceae, Chlorophyta, and Rhodymeniophycidae.

## Discussion

### Sponge Barcoding

To overcome the challenges of traditional taxonomy in the Aplysinidae family, DNA barcoding was employed (Wörheide and Erpenbeck, 2007). Analysis of the genomes from *A. fulva* and *A. cauliformis* revealed that the mitochondrial subunit I of the cytochrome C oxidase (cox-1) sequence presents low levels of variation and thus we could not separate these related species (Sperling et al., 2012). Instead, the internal transcribed spacer (ITS) was capable of differentiating several *Aplysina* species (Lamarão et al., 2010). Nevertheless, neither cox-1 nor the ITS were capable to differentiate the *Aplysina* species investigated here. Therefore, the cob, which has been efficient for separating new sponge species (Muricy et al., 2019), was applied here as well. Overall, the cob gene differentiated *A. caissara* and *A. fulva* collected along the coast of São Sebastião (São Paulo, Brazil) with good resolution. However, we were not able to distinguish other *Aplysina* species based on this gene, and caution should still be taken when using cob to identify *Aplysina* species. Even though our samples of *T. ignis* also formed a robust clade, the lack of cob sequences in the GenBank from other locations did not allow any further comparison.

### Holobiome Community Composition

This survey assessed for the first time the assembly processes as well as host-specificity and phylogeny relatedness of prokaryotic, fungal, and unicellular eukaryotic communities associated with three sympatric southwestern Atlantic sponges. We observed large differences in community diversity and composition across different species, as detected in previous work on different sponges (Hardoim et al., 2014; De Mares et al., 2017; Moitinho-Silva et al., 2017b; Steinert et al., 2017, 2019). Furthermore, we also observed that the sponge hosts showed a slightly lower community complexity than environmental samples, especially sediments, as shown in previous research (Thomas et al., 2016; Cleary et al., 2019; de Voogd et al., 2019). Several studies have shown that seawater did not reach a plateau when the dataset was not rarified (e.g., Thomas et al., 2016; de Voogd et al., 2019), whereas it seemed to be approaching the plateau for the prokaryotic community when the dataset was rarified (Cleary et al., 2019), which is consistent with our results (Supplementary Figure S2). Here, the fungal community in seawater was larger than that detected in other samples, whereas prokaryotic and unicellular eukaryotic communities were more abundant in sediments (Table 1). The microbial alpha ecological metrics of the three investigated communities in *A. caissara*, *A. fulva*, and *T. ignis* exhibited values between sediment and seawater samples. It is also noteworthy that phylogenetically closely related sponge species have dissimilar patterns of alpha ecological metrics, as observed in other host systems (Cadotte et al., 2019).

Strong evidence of a convergent pattern of evolution traits (i.e., *H'* and *D*_2_ diversity indices) between the structure of prokaryotic and fungal communities and host phylogenetic relatedness was shown. Similar results were obtained from bacterial communities associated with various sponge species (Rossi et al., 2011; Schöttner et al., 2013; Easson and Thacker, 2014). The prediction of *H'* and *D*_2_ diversity indices is strongly influenced by the evenness in the abundance of individuals within each taxon and by the relative abundance of the most common taxa in a community, respectively (Magurran, 2004). Therefore, the closely related sympatric Brazilian sponges revealed values similar to the predicted even abundance and the relative abundance of the most common prokaryotic and fungal taxa, but not in the unicellular eukaryotic species.

The number of prokaryotic phyla associated with sponges distributed from other locations than the southwestern Atlantic coast ranged from 41 to 72 (Thomas et al., 2016; Moitinho-Silva et al., 2017a). The same holds true for Brazilian *A. fulva* and *T. ignis*, whereas for *A. caissara* this value was lower. Despite the high number of sponge-associated prokaryotic phyla, the community composition was largely different for *Aplysina* species. For instance, the most abundant phyla encountered in *A. fulva*, *A. cauliformis*, *A. archeri*, *A. cavenicola*, and *A. aerophoba* sampled in seven distinct sites were assigned to Proteobacteria, Chloroflexi, unclassified bacteria, Acidobacteriota, and Actinobacteriota (Thomas et al., 2016), or *A. fulva* collected in the coast of Rio de Janeiro (Brazil) with the community dominated by Cyanobacteria, Proteobacteria, and Chloroflexi (Hardoim et al., 2009). The abundance of the phyla Chloroflexi, Acidobacteriota, Actinobacteriota, PAUC34f, Gemmatimonadota, Poribacteria, AncK6, Nitrospirota, and Spirochaetota and the classes Anaerolineae, Acidimicrobiia, PAUC34f, TK17, Poribacteria, AncK6, Nitrospiria, Rhodothermia, unclassified Chloroflexi, unclassified Actinobacteriota, and Spirochaetia are higher in HMA than LMA sponges (Moitinho-Silva et al., 2017b). These lineages were enriched in *Aplysina* species compared to *T. ignis*, which largely hosted unclassified bacteria and Gammaproteobacteria (Supplementary Table S4). These results emphasize the influence of a geographic niche in the assembly composition of prokaryotic community and that the *Aplysina* species are HMA sponges, whereas *T. ignis* might be characterized as an LMA sponge.

In addition to this geographic niche, the prokaryotic community associated with Brazilian sponges was strikingly distinct from the ones observed in environmental samples. For instance, between 47 and 70% of the prokaryotic community was exclusively associated with a single host. Thus, the differences in relative abundances and high taxonomic levels associated with these three sympatric species suggest that the host plays a pivotal role in shaping the structure of its own prokaryotic community. This is a pattern detected in many sponges collected in distinct places (Hardoim et al., 2014; Thomas et al., 2016; De Mares et al., 2017; Steinert et al., 2017, 2019).

The number of detected fungal phyla associated with *A. caissara*, *A. fulva*, and *T. ignis* in this study was higher than that for other sponge species (De Mares et al., 2017; Naim et al., 2017; Nguyen and Thomas, 2018). The most dominant phyla associated with several sponges collected in other locations, including *A. aerophoba* and *A. cauliformis*, were assigned to Ascomycota and Basidiomycota (He et al., 2014; De Mares et al., 2017; Naim et al., 2017; Nguyen and Thomas, 2018). In the present survey, these phyla together with unclassified fungi were the dominant phyla. Overall, in *Aplysina* species, Ascomycota ranged between approximately 50 and 70% (De Mares et al., 2017), whereas in the present study, Ascomycota associated with *A. caissara*, *A. fulva*, and *T. ignis* was limited to 23, 26, and 9% of the OTUs, respectively. Therefore, the investigated sponge species showed a more diverse composition than previously detected. Moreover, as showed for the prokaryotic community, the fungal community associated with the Brazilian sponges and detected in environmental samples were markedly distinct, with between 64.8 and 75.6% of the fungal community exclusively associated with a host. Overall, these results revealed that Brazilian sponge species form a strong host-fungal specificity bond.

Sediment was used for the first time to assess host-specificity and fungal community composition in comparison with host sponges. The only OTUs shared between sponge species and sediment were the same as the core and demonstrated a low influence of the sediment in the sponge fungal community. The difference between hosts and seawater was also observed in other species, including *A. aerophoba* and *A. cauliformis* (De Mares et al., 2017; Naim et al., 2017). Additionally, few studies have considered that the associated fungal community showed low host-specificity and even that their presence was rather “accidental” (Naim et al., 2017; Nguyen and Thomas, 2018). On the other hand, De Mares et al. (2017) argued that the fungal community was host-specific. The fungal community associated with Brazilian sponges was demonstrated to be host-specific (Figure 5). Moreover, in agreement with Nguyen and Thomas (2018), we also believe that the use of the ITS region instead of the 18S rRNA gene offers a more accurate assessment of fungal diversity associated with sponge species.

In this survey, 15 unicellular eukaryotic phyla were associated with *A. caissara*, *A. fulva*, and *T. ignis*, whereas 11 fungal and protist phyla were identified in 11 Chinese sponge species (He et al., 2014), six microbial eukaryotic phyla were also recorded in eight Antarctic sponge species (Rodríguez-Marconi et al., 2015), and 88 eukaryotic phyla were found in four marine sponges, including *A. aerophoba* and *A. cauliformis* collected from the Mediterranean and the Caribbean Seas, respectively (De Mares et al., 2017). However, the latter maintained macro-eukaryotes such as Arthropoda, Cnidaria, Mollusca, or Chordata in the dataset, which increased the number of phyla detected beyond the size of microorganisms. Three phyla, unclassified Eukaryota, Diatomea, and Florideophycidae dominated the microbial eukaryotic community associated with the Brazilian sponges. In Chinese sponges, the microbial eukaryotic phyla Ascomycota, Alveolata, and Chlorophyta were the most abundant (He et al., 2014). In the Antarctic sponges, Alveolata, Stramenopiles, and Hacrobia were the dominant phyla (Rodríguez-Marconi et al., 2015). Unclassified Eukaryota dominated the microbial eukaryotic assemblage associated with four sponge species, including *A. aerophoba* and *A. cauliformis* (De Mares et al., 2017). Thirty-two classes were detected in *A. caissara*, *A. fulva*, and *T. ignis*, and among them the most abundant were unclassified Eukaryota, Mediophyceae, and Nemaliophycidae, whereas from a total of 18 classes, the most abundant in Antarctic sponges were Syndiniales and Bacillariophyta (Rodríguez-Marconi et al., 2015). Furthermore, between 59 and 82% of the OTUs were exclusively associated with the investigated sponge species, whereas not a single OTU was common to sponge species and environmental samples. Our results demonstrate the strong specificity of the unicellular eukaryotic community associated with Brazilian sponge species.

Considering that the studies of He et al. (2014) and Rodríguez-Marconi et al. (2015) did not have biological replicates, and only the latter contained seawater to compare the community structure with sponge species, in addition to the apparently non-normalization of the data prior to alpha diversity metrics in both the studies and De Mares et al. (2017), a comparison among these studies and the present one is not appropriate. Previously, it was concluded that the unicellular eukaryotic community did not show host-specificity (De Mares et al., 2017). However, taking into account the results obtained here, the unicellular eukaryotic community associated with *A. caissara*, *A. fulva*, and *T. ignis* were host-specific and once again the host most likely plays a major role in selecting its symbionts. Interestingly, this host-specificity is not associated with the host phylogenetic relatedness, suggesting that a factor other than evolutionary influence is likely to be the main driving force for the selection of microbial eukaryotic community.

The three microbiome datasets contained an astonishing number of unclassified sponge-associated OTUs that ranged from 52 to 67% for prokaryotes and unicellular eukaryotes, respectively. For the prokaryotic dataset, 77% of these unclassified OTUs could not be classified to a species level (Supplementary Table S5). A similar value was also detected in the sympatric *A. caissara*, *Axinella corrugata*, and *Dragmacidon reticulatum* collected from a nearby region in São Sebastião (Hardoim et al., 2021). Around 69% of the unclassified fungal OTUs remains uncharacterized to a species level (Supplementary Table S11). This novelty value is even higher when replicates of *T. ignis* were removed due to low abundance of their samples (Supplementary Material). Around 80% of investigated unclassified lineages associated with *Aplysina* species revealed similarity below 97% against the NCBI ITS RefSeq Targeted Loci (RTL) database (Supplementary Table S25). When considering unicellular eukaryotes, around 53% of the unclassified OTUs associated with Brazilian sponges revealed similarity below 97% against the Silva database (Supplementary Table S16). Once again, when removing replicates with low abundance sequences (Supplementary Material), this novelty value reached more than 61% (Supplementary Table S32). Taken together, the Brazilian sponges encompassed an important reservoir of untapped microbial diversity.

### Deterministic Processes Dominated the Assembly of Sponge-Associated Microbial Community

Microbial species can be partitioned into habitat generalists and specialists on the basis of their distinct capacities to adapt to environmental challenges. While the former might easily thrive in a broader range of environmental conditions, the latter is often restricted to specific habitats due to their narrow environmental tolerances (Székely and Langenheder, 2014). This is important to model and predict the fate of segmented members of the community in the ecosystem, especially in the context of global changes (Muller, 2019). As expected, the assembly of habitat generalists and specialists is controlled by different ecological processes (Langenheder and Székely, 2011). For instance, water samples from lakes located on the Yungui Plateau (China) revealed that habitat specialists from the bacterial community were mainly governed by niche processes, whereas habitat generalists were strongly driven by neutral processes (Liao et al., 2016). Here, the assembly of habitat specialists inside the endemic *A. caissara* was partially shaped by neutral processes as compared to habitat generalists (Supplementary Figure S4), but not for *A. fulva* and *T. ignis*, which have a much larger distribution. The assembly of habitat generalists, independently of the host sponge species, was mainly driven by niche processes. Several studies have corroborated that the assemblage of sponge-associated bacterial microbiota is largely influenced by deterministic abiotic and biotic processes, including host signature, microbe-microbe interactions, and environmental conditions (Hardoim et al., 2014; De Mares et al., 2017; Steinert et al., 2017, 2019). Therefore, the magnitude of sponge species-dependent effect on the assemblage of bacterial community typically appears quite strong, which is consistent with our results (Figure 3). Interestingly, the assembly of whole fungal and habitat specialist unicellular eukaryotic communities associated with *T. ignis* was partially driven by stochasticity processes (Supplementary Tables S6B and S33B). It is tempting to speculate that the LMA-HMA dichotomy in sponge species is also governed by the assembly processes of microbial communities other than only prokaryotes. Therefore, more studies with the host holobiome are needed to shed more light onto this interesting topic.

Overall, the host-specificity observed in prokaryotic, fungal, and unicellular eukaryotic communities most likely contributes to fitness, resilience, and health of the sponge species, as has been observed in other systems. For instance, the specificity of the bacterial community associated with octocorals provided fitness and defense mechanisms for the host (van de Water et al., 2018), whereas the specific core bacteriome associated with three coral species demonstrated that it was mainly involved in improving the fitness (Hernandez-Agreda et al., 2017). The latter study also determined that in a reef the bacterial community might be taxonomically and, probably, functionally redundant within the coral host (Hernandez-Agreda et al., 2017). Furthermore, analyses of 25 ascidian species showed a high degree of host-specificity for prokaryotic members and that these communities promoted fitness and defense of the host ascidians (Erwin et al., 2014). Additionally, the bacterial communities associated with *Delisea pulchra*, a red seaweed, were demonstrated to be host-specific, stable, and resilient after a disturbance. The authors argued that when bacterial diversity is high it likely assists in the stability and enables enduring perturbations (Longford et al., 2019). Moreover, in human gut microbiota, stability suggested greater resilience of the host to disturbances (e.g., diseases and dysbiosis; Moya and Ferrer, 2016). On the other hand, fungal and microbial eukaryotic host-specificity was not detected in other systems, to the best of our knowledge. We believe that they are most likely involved in fitness, resilience, and health of the host sponges, however more studies are needed to verify this. Regarding the functional redundancy mentioned above, the holobiome associated with Brazilian sponges also encompassed lineages that are capable in performing the same function as discussed below.

### Lineages Responsible for Variability Detected

A majority of the prokaryotic phyla and few classes enriched in the three species have been detected in other marine sponges, including several *Aplysina*. Additionally, some of these phyla comprised several sponge-enriched clusters (Taylor et al., 2013; Hardoim et al., 2014, 2021; Thomas et al., 2016; Moitinho-Silva et al., 2017a; Bayer et al., 2018). Regarding prokaryotic lineages enriched in *A. caissara*, the genomic repertoire of symbionts associated with *A. aerophoba* revealed that Actinobacteriota, Acidobacteriota, Chloroflexi, Deinoccocota, Nitrospinota, and Spirochaetota had enriched clusters of orthologous groups (COGs) correlated with restriction-modification, which plays an essential role in defense against incoming foreign DNA and in symbioses (Slaby et al., 2017). Some of the metabolism features of Chloroflexi associated with *A. aerophoba* were glycolysis, carbon fixation, nitrogen cycling, biosynthesis of amino acids and cofactors, fatty acids biosynthesis and degradation, and potential aromatic degradation (Bayer et al., 2018). Halogenases were detected in Chloroflexi, Actinobacteriota, Acidobacteriota, and Spirochaetota associated with five *Aplysina* species and related to the brominated compounds production (Gutleben et al., 2019). In our study, unclassified Chloroflexi and unclassified Myxococcota will most likely encompass new species and possibly new functional capabilities. Thus, it is tempting to speculate that these functions are performed by the lineages enriched in *A. caissara*.

Considering the lineages enriched in *A. fulva*, in the metabolic reconstruction of the unicellular eukaryotes metagenomes of *A. aerophoba* and *Petrosia ficiformis*, PAUC34f contained genes involved in glycolysis and oxidative phosphorylation and that encoded numerous enzymes involved in the uptake and/or metabolism of nitrogen and sulfate and biosynthesis of amino acids, vitamins, purines, and pyrimidines (Astudillo-García et al., 2018). TK-10 contained halogenases, which are an important enzyme in the brominated and chlorinated secondary metabolites biosynthesis (Gutleben et al., 2019). These compounds are well known in *Aplysina* species (Lira et al., 2011) and provide protection against predation (Loh and Pawlik, 2014). However, it was demonstrated that Dehalococcoidia might be able to deactivate the chemical defense systems by dehalogenating halogenated signaling molecules (Yang et al., 2020). Therefore, these functions might be executed by the enriched lineages associated with *A. fulva*.

Three lineages enriched in *T. ignis* could only be assigned to the phylum level (bacteria, Actinobacteriota, and Proteobacteria), which will most likely reveal novel members and metabolic capabilities. The genomic repertoire of Holophagae associated with *A. aerophoba* was enriched in COGs related to metabolism and energy production (Slaby et al., 2017). One metagenome-assembled genome (MAG) affiliated to Desulfobacteria was obtained from *Cinachyrella* sp. and showed that the most abundant KEGG pathways were related to purine metabolism, ABC transporters, oxidative phosphorylation, aminoacyl-tRNA biosynthesis, and glyoxylate and dicarboxylate metabolisms. It also contained genes involved in sulfur metabolism and quorum sensing (Shmakova, 2020). Hence, a higher degree of most likely novel members and capabilities were detected in *T. ignis* compared with *Aplysina* species. Furthermore, it is tempting to speculate that the functions described above are performed by the lineages enriched in *T. ignis*.

For fungi, Microbotryomycetes and Ascomycota were enriched in *A. caissara* and *T. ignis*, respectively. The class Microbotryomycetes was detected as associated with several sponge species including *A. cauliformis* and *A. aerophoba* (De Mares et al., 2017; Naim et al., 2017; Nguyen and Thomas, 2018). Even though this class has been associated with sponges, no putative function of marine Microbotryomycetes was found. Thus, there is still a lot to be discovered regarding the functions that these symbionts are able to perform for the host.

For unicellular eukaryotic symbionts, the lineages Ascomycota, Basidiomycota, and Malasseziomycetes were enriched in *A. fulva*, whereas unclassified Eukaryotic and Floridiophycidae were enriched in *A. caissara*. Phylum Basidiomycota has been detected in several sponges collected from other locations (He et al., 2014; Rodríguez-Marconi et al., 2015; De Mares et al., 2017; Naim et al., 2017; Nguyen and Thomas, 2018). Thirteen Basidiomycota were isolated from several species, including *A. aerophoba*, and assigned putative functions, such as cytotoxic activities, biosurfactant producers, capacity to degrade polycyclic aromatic hydrocarbons, protective role against pathogens, and/or damage caused by potential mutagenic compounds (Poli et al., 2018). The class Malasseziomycetes encompasses one genus (*Malassezia*; Jones et al., 2015) and it has been detected in several marine sponges, including *A. aerophoba* (Naim et al., 2017; Nguyen and Thomas, 2018). It is tempting to speculate that the functions mentioned above are likely to be performed by the lineages enriched in *A. fulva*.

The unclassified Eukaryotic enriched in *A. caissara* will most likely represent novel species and possibly new functions that are waiting to be discovered, considering that 38 OTUs from a total of 48 were host-specific. The same holds true for Floridiophycidae, where all the OTUs detected in *A. caissara* were specific. No putative function of Floridiophycidae associated with marine sponge was recovered, and thus no conclusion could be drawn at the moment regarding the putative roles this lineage plays for the host sponge.

Ascomycota were enriched in *T. ignis* within the fungi dataset and in *A. fulva* within the unicellular Eukaryota dataset. All the fungi isolated from marine sponges that exhibited antimicrobial activities were affiliated to Ascomycota (Indraningrat et al., 2016). In the sponge *Grantia compressa*, 77.8% of the fungi isolated were affiliated to Ascomycota. Among them, one strain produced 10 secondary metabolites and some of them exhibited antimicrobial and antiviral activities, and antifouling (Bovio et al., 2019a,b). One Ascomycota strain isolated from mid-Atlantic San Peter and San Paul Archipelago *A. fulva* produced secondary metabolites with antibacterial activities (Martins et al., 2021). It is tempting to speculate that Ascomycota members might be responsible for the defense of the host sponges.

## Conclusion

The holobiome associated with *A. caissara*, *A. fulva*, and *T. ignis* was drastically different from the ones obtained in the environmental samples. Between 47 and 82% of the OTUs in the prokaryotic, fungal, and unicellular eukaryotic datasets were specifically associated with the three sympatric sponge species. Moreover, it was shown for the three datasets analyzed here that the Brazilian sponges represent a reservoir of novel microbial diversity, with closely related sponges tending to harbor a similar pattern of microbial community structure. In addition to host-specificity and novelty, we also observed that niche processes are the dominant force influencing the holobiome assembly of *Aplysina* species, while the assembly of whole fungal and habitat specialist unicellular eukaryotic communities in *T. ignis* is also influenced by neutral processes. Furthermore, phylogenetic signal analysis revealed that ecological traits of prokaryotic and fungal communities associated with *A. caissara*, *A. fulva*, and *T. ignis* were also host-related, whereas the unicellular eukaryotic community was only host identity-specific. Most of the species-rich sponge-associated lineages documented in this study are also observed in other sponge species collected in the Northern seas. Many of them might also play essential roles in the symbioses, including glycolysis, carbon fixation, nitrogen cycling, and biosynthesis of secondary metabolites that exhibited antimicrobial and antiviral activities as well as provide protection against host predation. Overall, it seems that the sponge species play a major role in picking their own associated holobiome.

## Permits

Sampling was performed under the scientific collection permits A097B99 issued by Sistema Nacional de Gestão do Patrimônio Genético e do Conhecimento Tradicional Associado, 61460-2 issued by Sistema de Autorização e Informação sobre Biodiversidade do Instituto Chico Mendes de Conservação da Biodiversidade, both from the Ministério do Meio Ambiente and 260108-001.161/2013 issued by the Instituto Florestal, Secretaria do Meio Ambiente do Estado de São Paulo.

## Data Availability Statement

The datasets presented in this study can be found in online repositories. The names of the repository/repositories and accession number(s) can be found in the article/Supplementary Material.

## Author Contributions

CH designed the experiment, collected and pre-processed the samples, prepared the samples for high-throughput sequencing, performed the data analysis, and wrote the manuscript with feedback from all co-authors. MC and GL-H identified the sponge species. PH performed the statistical analyses. All authors contributed to the article and approved the submitted version.

### Conflict of Interest

The authors declare that the research was conducted in the absence of any commercial or financial relationships that could be construed as a potential conflict of interest.

## Footnotes

^1^https://github.com/GuillemSalazar/EcolUtils

^2^http://jvenn.toulouse.inra.fr/app/example.html

## Supplementary Material

Supplementary Figure S1 The Maximum Likelihood tree (−ln likelihood:6296.906436) of the Aplysinidae and Tedaniidae families based on the cytochrome b is shown. ML bootstrap values (>75) and Bayesian posterior probabilities (>0.90) are shown above and below branches, respectively.

Supplementary Figure S2 Rarefaction curves of prokaryotic community for Ac (*Aplysina caissara*), Af (*Aplysina fulva*), Ti (*Tedania ignis*), SW (seawater), and SD (sediment).

Supplementary Figure S3 Community composition of habitat generalist and specialist for prokaryotic datasets are shown for phylum **(A,C)** and class **(B,D)**, respectively. Ac (*Aplysina caissara*), Af (*Aplysina fulva*), Ti (*Tedania ignis*), SW (seawater), and SD (sediment).

Supplementary Figure S4 Neutral model applied to assess the effect of random dispersion and ecological drift on the assembly of *Aplysina caissara* **(A)**, *Aplysina fulva* **(B)**, *Tedania ignis* **(C)**, and sediment **(D)** for prokaryotic community.

Supplementary Figure S5 Phylogenetic tree of the prokaryotic habitat generalist containing OTUs at the branch’s tips. The inference was obtained with Maximum Likelihood with 100 bootstraps and GTR + I + G model, and visualized and edited using the interactive tree of life (iTol; Letunic and Bork, 2019) tool. Ac (*Aplysina caissara*), Af (*Aplysina fulva*), Ti (*Tedania ignis*), SW (seawater), and SD (sediment).

Supplementary Figure S6 Phylogenetic tree of the prokaryotic habitat specialist containing OTUs at the branch’s tips. The inference was obtained with Maximum Likelihood with 100 bootstraps and GTR + I + G model, and visualized and edited using the interactive tree of life (iTol; Letunic and Bork, 2019) tool. Ac (*Aplysina caissara*), Af (*Aplysina fulva*), Ti (*Tedania ignis*), SW (seawater), and SD (sediment).

Supplementary Figure S7 Community composition for the habitat generalist and specialist groups are shown for fungal **(A,B)** and unicellular eukaryotes **(C,D)** datasets, respectively. Ac (*Aplysina caissara*), Af (*Aplysina fulva*), Ti (*Tedania ignis*), SW (seawater), and SD (sediment).

Supplementary Figure S8 Rarefaction curves of fungal **(A)** and unicellular eukaryotic **(B)** community for Ac (*Aplysina caissara*), Af (*Aplysina fulva*), Ti (*Tedania ignis*), SW (seawater), and SD (sediment).

Supplementary Figure S9 Rarefaction curves of fungal community for Ac (*Aplysina caissara*), Af (*Aplysina fulva*), SW (seawater), and SD (sediment) **(A)** unicellular eukaryotic community for Ac, Ti (*Tedania ignis*), SW, and SD **(B)**.

Supplementary Figure S10 Non-metric multidimensional scaling (nMDS) based on Bray–Curtis distances for the whole **(A)**, habitat generalist **(B)**, and habitat specialist **(C)** fungal groups, and whole **(D)**, and habitat specialist **(E)** unicellular eukaryotic groups. Ac (*Aplysina caissara*), Af (*Aplysina fulva*), Ti (*Tedania ignis*), SW (seawater), and SD (sediment).

Supplementary Figure S11 Fungal community composition for Ac (*Aplysina caissara*), Af (*Aplysina fulva*), SW (seawater), and SD (sediment) are shown for 10 phyla **(A)** and for 31 classes **(B)**.

Supplementary Figure S12 Community composition of habitat generalist and specialist groups for fungal dataset are shown for phylum **(A,C)** and class **(B,D)**, respectively. Ac (*Aplysina caissara*), Af (*Aplysina fulva*), SW (seawater), and SD (sediment).

Supplementary Figure S13 Venn diagram with all OTUs detected in Ac (*Aplysina caissara*, magenta), Af (*Aplysina fulva*, green), SW (seawater, blue), and SD (sediment, brown), and taxonomic representation of statistically and biologically consistent differences across Ac, Af, SW, and SD are presented for the fungal **(A,C)** dataset and Venn diagram with all OTUs detected in Ac (magenta), *Tedania ignis* (Ti, red), SW (blue), and SD (brown), and taxonomic representation of statistically and biologically consistent differences across Ac, Ti, SW, and SD for the unicellular eukaryotic **(B,D)** dataset.

Supplementary Figure S14 Phylogenetic tree of the fungi habitat generalist containing OTUs at the branch’s tips. The inference was obtained with Maximum Likelihood with 100 bootstraps and GTR + I + G model, and visualized and edited using the interactive tree of life (iTol; Letunic and Bork, 2019) tool. Ac (*Aplysina caissara*), Af (*Aplysina fulva*), SW (seawater), and SD (sediment).

Supplementary Figure S15 Phylogenetic tree of the fungi habitat specialist containing OTUs at the branch’s tips. The inference was obtained with Maximum Likelihood with 100 bootstraps and GTR + I + G model and visualized and edited using the interactive tree of life (iTol; Letunic and Bork, 2019) tool. Ac (*Aplysina caissara*), Af (*Aplysina fulva*), SW (seawater), and SD (sediment).

Supplementary Figure S16 Unicellular eukaryotic community composition for Ac (*Aplysina caissara*), Ti (*Tedania ignis*), SW (seawater), and SD (sediment) are shown for 27 phyla **(A)** and for 42 classes with relative abundance >0.02% **(B)**.

Supplementary Figure S17 Community composition of habitat specialist for unicellular eukaryotic dataset are shown for phylum **(A)** and class **(B)**. Ac (*Aplysina caissara*), Ti (*Tedania ignis*), SW (seawater), and SD (sediment).

Supplementary Table S1 OTUs vs. samples table containing all categories and OTU taxonomic assignments for the prokaryotic dataset.

Supplementary Table S2 Statistical analyses of the alpha ecological metrics for **(A)** prokaryotic, **(B)** fungal, and **(C)** unicellular eukaryotic datasets. Ac: *Aplysina caissara*, Af: *Aplysina fulva*, Ti: *Tedania ignis*, SW: seawater, SD: sediment are shown.

Supplementary Table S3 Permutational multivariate analysis of variance (PERMANOVA) and analysis of similarity (ANOSIM) across different datasets.

Supplementary Table S4 Relative abundance of the prokaryotic composition for phylum **(A)** and class **(B)**. Ac: *Aplysina caissara*, Af: *Aplysina fulva*, Ti: *Tedania ignis*, SW: seawater, SD: sediment.

Supplementary Table S5 Results from BLASTn of the prokaryotic unclassified OTUs associated with sponge species **(A)** and summary of the taxonomic distribution of the unclassified OTUs **(B)** are shown. Ac: *Aplysina caissara*, Af: *Aplysina fulva*, Ti: *Tedania ignis*, SW: seawater, SD: sediment.

Supplementary Table S6 Pairwise test of significance of the modified stochasticity ratio (MST) across different datasets based on permutational multivariate analysis of variance (PERMANOVA) **(A)** and Sloan null hypothesis of sponge microbial community assembly **(B)**. Ac: *Aplysina caissara*, Af: *Aplysina fulva*, Ti: *Tedania ignis*, SW: seawater, SD: sediment.

Supplementary Table S7 Prokaryotic taxonomic composition of OTUs obtained exclusively in *Aplysina caissara* **(A)**, *Aplysina fulva* **(B)**, *Aplysina* species **(C)**, *Tedania ignis* **(D)**, sponge core **(E)**, seawater **(F)**, sediment **(G)**, and the core of all categories **(H)**.

Supplementary Table S8 List of enriched features provided by linear discriminant analysis (LDA) of effect size (LEfSe) that are differential across sponge species, seawater, and sediment with statistical and biological significance, and ranked according to the effect size (LDA) for whole **(A)**, habitat generalist **(B)**, and habitat specialist **(C)** prokaryotic groups.

Supplementary Table S9 OTUs vs. samples table containing all categories and OTU taxonomic assignments for the fungal dataset.

Supplementary Table S10 Relative abundance of the fungi composition for phylum **(A)** and class **(B)**. Ac: *Aplysina caissara*, Af: *Aplysina fulva*, Ti: *Tedania ignis*, SW: seawater, SD: sediment.

Supplementary Table S11 Results from BLASTn of the fungal unclassified OTUs associated with sponge species **(A)** and summary of the taxonomic distribution of the unclassified OTUs **(B)** are shown. Ac: *Aplysina caissara*, Af: *Aplysina fulva*, Ti: *Tedania ignis*, SW: seawater, SD: sediment.

Supplementary Table S12 Fungal taxonomic composition of OTUs obtained exclusively in *Aplysina caissara* **(A)**, *Aplysina fulva* **(B)**, *Tedania ignis* **(C)**, seawater **(D)**, sediment **(E)**, and the core of all categories **(F)**.

Supplementary Table S13 List of enriched features provided by linear discriminant analysis (LDA) of effect size (LEfSe) that are differential across sponge species, seawater, and sediment with statistical and biological significance, and ranked according to the effect size (LDA) for the whole fungal community.

Supplementary Table S14 OTUs vs. samples table containing all categories and OTU taxonomic assignments for the unicellular eukaryotic dataset.

Supplementary Table S15 Relative abundance of the unicellular eukaryotic composition for phylum **(A)** and class **(B)**. Ac: *Aplysina caissara*, Af: *Aplysina fulva*, Ti: *Tedania ignis*, SW: seawater, SD: sediment.

Supplementary Table S16 Results from BLASTn of the unicellular eukaryotic unclassified OTUs associated with sponge species **(A)** and summary of taxonomic distribution of the unclassified OTUs **(B)** are shown. Ac: *Aplysina caissara*, Af: *Aplysina fulva*, Ti: *Tedania ignis*, SW: seawater, SD: sediment.

Supplementary Table S17 Unicellular eukaryotic taxonomic composition of OTUs obtained exclusively in *A. caissara* **(A)**, *Aplysina fulva* **(B)**, *Aplysina ignis* **(C)**, sponge core **(D)**, seawater **(E)**, and sediment **(F)**.

Supplementary Table S18 List of enriched features provided by linear discriminant analysis (LDA) of effect size LEfSe that are differential across sponge species, seawater, and sediment with statistical and biological significance, and ranked according to the effect size (LDA) for the whole the unicellular eukaryotic group.

Supplementary Table S19 OTUs vs. samples table containing all categories and OTU taxonomic assignments for fungal dataset.

Supplementary Table S20 Fungal community composition of the habitat generalist group **(A)** and habitat specialist group **(B)**. Ac: *Aplysina caissara*, Af: *Aplysina fulva*, SW: seawater, SD: sediment.

Supplementary Table S21 Values for richness, diversity, and evenness indices. Ac: *Aplysina caissara*, Af: *Aplysina fulva*, Ti: *Tedania ignis*, SW: seawater, SD: sediment.

Supplementary Table S22 Statistical analyses of the alpha ecological metrics for the fungal dataset **(A)** and for the unicellular eukaryotic dataset **(B)**. Ac: *A. caissara*, Af: *Aplysina fulva*, Ti: *Tedania ignis*, SW: seawater, SD: sediment.

Supplementary Table S23 Permutational multivariate analysis of variance (PERMANOVA) and analysis of similarity (ANOSIM) of whole, habitat generalist, and specialist groups of fungi and unicellular eukaryotic.

Supplementary Table S24 Relative abundance of the fungal composition for phylum **(A)** and class **(B)**. Ac: *Aplysina caissara*, Af: *Aplysina fulva*, SW: seawater, SD: sediment.

Supplementary Table S25 Results from BLASTn of the fungal unclassified OTUs associated with sponge species **(A)** and summary of the taxonomic distribution of the unclassified OTUs **(B)** Ac: Aplysina caissara, Af: Aplysina fulva.

Supplementary Table S26 Stochastic ratio of fungi community assembly estimated by modified stochasticity ratio (MST) analysis with Bray–Curtis dissimilarity distance **(A)** and Sloan null hypothesis of fungal community assembly **(B)**. Ac: *Aplysina caissara*, Af: *Aplysina fulva*, SW: seawater, SD: sediment.

Supplementary Table S27 Fungal taxonomic composition of OTUs obtained exclusively in *Aplysina caissara* **(A)**, *Aplysina fulva* **(B)**, *Aplysina* species **(C)**, seawater **(D)**, sediment **(E)**, and the core of all categories **(F)**.

Supplementary Table S28 List of enhanced features provided by linear discriminant analysis (LDA) of effect size LEfSe that are differential across sponge species, seawater, and sediment with statistical and biological significance, and ranked according to the effect size (LDA) for the whole fungal **(A)**, for habitat generalist fungal **(B)**, and for habitat specialist **(C)** groups.

Supplementary Table S29 OTUs vs. samples table containing all categories and OTU taxonomic assignments for the unicellular eukaryotes dataset.

Supplementary Table S30 Unicellular eukaryotic community composition of the habitat generalist group **(A)** and habitat specialist group **(B)**. Ac: *Aplysina caissara*, Ti: *Tedania ignis*, SW: seawater, SD: sediment.

Supplementary Table S31 Relative abundance of the unicellular eukaryotic composition for phylum **(A)** and class **(B)**. Ac: *Aplysina caissara*, Ti: *Tedania ignis*, SW: seawater, SD: sediment.

Supplementary Table S32 Results from BLASTn of the unicellular eukaryotic unclassified OTUs associated with sponge species **(A)** and summary of the taxonomic distribution of the unclassified OTUs **(B)**. Ac: *Aplysina caissara*, Ti: *Tedania ignis*.

Supplementary Table S33 Stochastic ratio of unicellular eukaryotic community assembly estimated by modified stochasticity ratio (MST) analysis with Bray–Curtis dissimilarity distance **(A)** and Sloan null hypothesis of unicellular eukaryotic community assembly **(B)**. Ac: *Aplysina caissara*, Af: *Aplysina fulva*, SW: seawater, SD: sediment.

Supplementary Table S34 Unicellular Eukaryotic taxonomic composition of OTUs obtained exclusively in *Aplysina caissara* **(A)**, *Tedania ignis* **(B)**, sponge species **(C)**, seawater **(D)**, sediment **(E)**, and the core of all categories **(F)**.

Supplementary Table S35 List of enhanced features provided by linear discriminant analysis (LDA) of effect size LEfSe that are differential across sponge species, seawater, and sediment with statistical and biological significance, and ranked according to the effect size (LDA) for the whole unicellular eukaryotic **(A)** and for habitat specialist unicellular eukaryotic **(B)** groups.
