# Supplementary material for: Prokaryotic, Fungal, and Unicellular Eukaryotic Core Communities Across Three Sympatric Marine Sponges From the Southwestern Atlantic Coast Are Dominated Largely by Deterministic Assemblage Processes
Source: Front Microbiol. 2021 Jun 8;12:674004. doi: 10.3389/fmicb.2021.674004 (PMC8217869; doi:10.3389/fmicb.2021.674004)
Supplement: Supplementary file 1 [file Data_Sheet_1.zip › data sheet 4/supplementary material_vf.doc]

**Prokaryotic, Fungal, and Unicellular Eukaryotic Core Communities across Three Sympatric Marine Sponges from the Southwestern Atlantic Coast are Dominated largely by Deterministic Assemblage Processes**

Cristiane C. P. Hardoim^*^; Gisele Lôbo-Hajdu; Márcio R. Custódio; Pablo R. Hardoim

***Corresponding author.** E-mail cristianehardoim@gmail.com

***Illumina sequencing***

An aliquot of the purified genomic DNA was submitted to Functional Genomics Center of the Luiz de Queiroz College of Agriculture (ESALQ-USP) to perform 16S and 18S rRNA genes as well as the internal transcribed spacer (ITS) of the ribosomal RNA gene operon sequencing following all appropriate quality controls. Briefly, the V4-region 16S rRNA gene, targeting the prokaryotic community, was amplified with the primer pair 515F (5´-GTGYCAGCMGCCGCGGTAA-3´, Parada et al., 2016) and 806R (5´-GGACTACNVGGGTWTCTAAT-3´, Apprill et al., 2015). For the ITS, the primer pair used was ITS1f (5’-CTTGGTCATTTAGAGGAAGTAA-3’) and ITS2 (5’-GCTGCGTTCTTCATCGATGC-3’) (Gardes and Bruns, 1993; Smith and Peay, 2014) targeting the fungal community. The V9-region 18S rRNA gene, aiming the microbial eukaryotic community, was amplified with primer pair Euk1391f (5´-GTACACACCGCCCGTC-3´, Amaral-Zettler et al., 2009) and EukBr (5’-TGATCCTTCTGCAGGTTCACCTAC-3’, Stoeck et al., 2010). The reaction mixture (25 μl) encompassed of 2.5 μl of template DNA (20 ng μl^-1^) for 16S rRNA gene and ITS or of 1.0 μl of template DNA (20 ng μl^-1^) for 18S rRNA, 0.20 mM of each primer, and 2X PCRBio Ultra Mix (PCRBiosystems, Wayne, USA). The thermal cycle for 16S rRNA gene started with 3 min at 95°C, followed by 30 cycles of 30 sec at 95°C, 30 sec at 60°C, and 30 sec at 72°C were carried out. A final extension of 10 min at 72°C was applied to complete the reaction. For the ITS, the thermal cycle was composed of 85 sec at 94°C, followed by 35 cycles of 35 sec at 94°C, 55 sec at 64°C, and 45 sec at 72°C were performed. A final extension of 10 min at 72°C was used for reaction completion. Whereas for 18S rRNA gene the thermal cycle consisted of 3 min at 94°C, followed by 30 cycles of 30 sec at 94°C, 30 sec at 57°C, and 30 sec at 72°C. A final extension of 10 min at 72°C was used to complete the reaction. These amplicons were subjected to Illumina sequencing using MiSeq platform.

***Illumina sequencing analyses***

Illumina sequences were processed using Mothur v. 1.44 (Schloss et al., 2009). For each dataset (16S and 18S rRNA genes and ITS), a pipeline was optimized and executed. Thus, for the 16S rRNA gene initially, the command make.contigs was used to join the paired raw sequences. Then, the demultiplexed fastq sequences had the first quality control using the command screen.seqs with parameters: fasta=, group=, maxambig=0, maxhomop=8, minlength=270, maxlength=320. The commands unique.seqs and count.seqs were used to reduce the dataset to non-identical sequences and thus minimize computational effort. Prior to the alignment of the non-redundant sequences, the positions at which the primer pair aligned in the 16S rRNA gene was searched using a sequence of *Escherichia coli* (J01851) and the reference SILVA seed v. 138 database (mothur-formatted), provided by Mothur (Quast et al., 2013) with the command align.seqs and parameters fasta=ecoli.fasta, reference=silva.seed_v138.align. The positions that the reference database should be trimmed were shown by the command summary.seqs. Then the command pcr.seqs was carried out with parameters fasta=silva.seed_v138.align, start=11895, end=25318, keepdots=F and then aligned with align.seqs command and parameters fasta=, reference=silva.seed_v138.pcr.align. The sequences aligned to the expected position were maintained (screen.seqs: fasta=, count=, start=2, end=13423, criteria=90 and filtered with filter.seqs: fasta=, vertical=T, trump=.). Aligned sequences were reduced to non-redundant sequences with the command unique.seqs. Prior to chimera check, the sequences were pre-clustered (pre.cluster: fasta=, count=, diffs=2), which allowed up to two differences between sequences. Then, chimeric sequences were detected with UCHIME (Edgar et al., 2011) (chimera.uchime command with parameters: fasta=, count=, dereplicate=t) and filtered out with the command remove.seqs. The command pcr.seqs was applied again to trim the aligned SILVA non-redundant v. 138 database (Quast et al., 2013; Yilmaz et al., 2014) with the parameters fasta=silva.nr_v138.align, start=11895, end=25318, keepdots=F. Then, sequences were phylogenetically classified with the command classify.seqs with the parameters fasta=, count=, taxonomy=silva.nr_v138.tax, reference=silva.nr_v138.pcr.align, cutoff=80. The undesirable sequences; classified as mitochondria, chloroplast, eukaryota, and unknown; were filtered out with the command remove.lineage. Then, the commands cluster.split with parameters: fasta=, count=, taxonomy=, taxlevel=4, splitmethod=classify, cutoff=0.03, method=average, cluster=f were applied. This script also calculated the pairwise distances between aligned sequences (dist.seqs: fasta=, cutoff=0.03). The output was used in cluster.split(file=). To filter the list file generated in the previous script, the command remove.rare(list=, count=, nseqs=1) was used, which removed singletons from the dataset. To list the sequences in the filtered file the command list.seqs(list=) was applied, followed by get.seqs(fasta=, taxonomy=, count=, accnos=) selecting filtered sequences from all files. Then, the command make.shared(list=, count=, label=0.03), where the shared file contained the Operational Taxonomic Units (OTUs) classified at 97% sequence similarity. The libraries were normalized with the command sub.sample(shared=). In order to get the representative sequences from each OTU, the command get.oturep(list=, fasta=, count=, method=abundance) was applied. OTUs were further classified based on the SILVA non-redundant v. 138 database (mothur-formatted) (Quast et al., 2013; Yilmaz et al., 2014) with the commands summary.tax(taxonomy=, count=), and classify.otu(list=, count=, taxonomy=, label=0.03).

For the analyses of ITS, the instructions from <https://github.com/krmaas/bioinformatics/blob/master/mothur.fungal.batch> were followed with few modifications. The command make.contigs was primarily used to unite the paired raw sequences. Afterwards, the command screen.seqs was applied in the demultiplexed fastq sequences with the parameters fasta=, group= summary=, maxambig=0, maxhomop=8, minlength=190, maxlength=400 to verify their quality. The commands unique.seqs and count.seqs were carried out as explained for the 16S rRNA gene dataset. Then, the reads were pre-clustered as abovementioned. The chimeric sequences were detected with UCHIME (Edgar et al., 2011) and performed as explained above, and filtered out with the command remove.seqs. For the classification of the reads, the UNITE v8.2 dataset, mothur-formatted (Abarenkov et al., 2020) was used with the command classify.seqs and parameters fasta=, count=, taxonomy=UNITEv8.2_sh_dynamic_s_all.tax, reference= UNITEv8.2_sh_dynamic_s_all.fasta, cutoff=60. The undesirable sequences; classified as Protista, Metazoa, Viridiplantae, Stramenopila, Rhizaria, and unknown; were filtered out with the command remove.lineage. The command cluster.split(fasta=, count=, cutoff=0.03, method=agc) was used to cluster the sequences. The command remove.rare(list=, count=, nseqs=1), which removed singletons from the dataset, was used to filter the list file generated in the previous script. Then the command list.seqs(list=) was carried out to list the sequences that were previously filtered, which was followed by get.seqs(fasta=, taxonomy=, count=) selecting filtered sequences from all files. The command make.shared(list=, count=, label=0.03) created the shared file encompassed by the OTUs classified at 97% sequence similarity. The command sub.sample(shared=) was used to normalize the libraries. In order to get the representative sequences from each OTU, the command get.oturep(list=, fasta=, count=, method=abundance) was applied. OTUs were further classified based on the UNITEv8.2_sh_dynamic_s_all.tax (Abarenkov et al., 2020) with commands summary.tax(taxonomy=, count=) and classify.otu(list=, count=, taxonomy=, label=0.03).

For 18S rRNA gene a similar approach as for 16S rRNA gene analyses was carried out. Firstly, the paired raw sequences were united with the command make.contigs. Then, the demultiplexed fastq sequences had the quality control checked with the command screen.seqs with parameters: fasta=, group=, maxambig=0, maxhomop=8, minlength=140, maxlength=310. The commands unique.seqs and count.seqs were performed as explained for the 16S rRNA gene dataset. Prior the use of the SILVA seed v. 138 database (Quast et al., 2013) provided by mothur, a sequence of *Saccharomyces cerevisiae* (J01353) was used to determine the positions of the primer pair in the 18S rRNA gene with the command align.seqs and parameters fasta=scerevisiae.fasta and reference=silva.seed_v138.align. The command summary.seqs revealed the positions that the reference database should be trimmed. Then, the command pcr.seqs was applied with parameters fasta=silva.seed_v138.align, start=41791, end=43116, keepdots=F and sequences were aligned with the command align.seqs and parameters: fasta=, reference=silva.seed_v138.pcr.align. The sequences that were aligned to the expected position were maintained (screen.seqs: fasta=, count=, start=3, end=1325, criteria=90) and then sequences were filtered with command filter.seqs(fasta=, vertical=T, trump=.). Aligned reads were reduced to non-redundant sequences with the command unique.seqs. The reads were pre-clustered as previously described. Then, UCHIME (Edgar et al., 2011) was used to detect chimeric sequences (chimera.uchime: fasta=, count=, dereplicate=t) and filtered out with the command remove.seqs. Prior to the classification of the sequences, the SILVA non-redundant v. 138 aligned database, mothur-formatted (Quast et al., 2013; Yilmaz et al., 2014) was trimmed with the command pcr.seqs and parameters fasta=silva.nr_v138.align, start=41791, end=43116, keepdots=F. Then, sequences were phylogenetically classified with the command classify.seqs and parameters fasta=, count=, taxonomy=silva.nr_v138.pick.tax, reference=silva.nr_v138.pcr.align, cutoff=60. The undesirable sequences; classified as Porifera, Bacteria, Archaea, Animalia, Annelida, Arthropoda, Cephalochordata, Cnidaria, Ctenophora, Echinodermata, Entoprocta, Hemichordata, Lophophorata, Mollusca, Nematoda, Nematozoa, Nemertea, Platyhelminthes, Rotifera, Tunicata, Vertebrata, and unknown; were filtered out with command remove.lineage. Then, pairwise distance and cluster.split were performed as detailed above. The following commands, remove.rare, list.seqs, get.seqs, make.shared, sub.sample, and get.oturep were performed as explained above. OTUs were further classified based on the SILVA non-redundant v. 138 database (Quast et al., 2013; Yilmaz et al., 2014) with the commands summary.tax(taxonomy=, count=) and classify.otu(list=, count=,taxonomy=, label=0.03).

***Calculation of community metrics***

For all datasets, the rarefaction curves were calculated within mothur with the command rarefaction.single and parameters: shared=, freq=1000 for 16S rRNA gene, freq=30 for 18S rRNA gene and freq=50 for ITS.

***Scripts for BLASTn analyses***

The following scripts were performed in the terminal for the taxonomic classification of sequences:

*Prokaryotic dataset*

Run BLASTn against SILVA database v. 138

blastn -query proka_novelty.fasta -db /home/dell-ubuntu/Documents/Databases/Silva_138 -max_target_seqs 1 -evalue 1e-20 -outfmt '6 qseqid sseqid pident evalue'-out proka_novelty_Silva.txt -num_threads 4

*Fungal dataset*

Run BLASTn against ITS ref seq

blastn -query fungi_novelty.fasta -db /home/dell-ubuntu/Documents/Databases/ITS_RefSeq_Fungi -max_target_seqs 1 -evalue 1e-20 -outfmt '6 qseqid sseqid pident evalue' -out fungi_novelty_ITS.txt -num_threads 4

*Unicellular Eukaryota dataset*

Run BLASTn against SILVA database

blastn -query euka_novelty.fasta -db /home/dell-ubuntu/Documents/Databases/Silva_138 -max_target_seqs 1 -evalue 1e-20 -outfmt '6 qseqid sseqid pident evalue' -out euka_novelty_Silva.txt -num_threads 4

**Results**

**EUKARYOTIC COMMUNITY COMPOSITION**

***Fungal dataset***

Samples from *Tedania ignis* showed low abundance in the fungal dataset and therefore, these samples were removed to enhance sequence depth of the overall data. By removing these samples, the original dataset with 958 OTUs (presented in the main text) was largely improved and the new dataset is now comprehended by 5,028 OTUs. Indeed, a total of 1,591,906 ITS region sequences were acquired. After denoising, quality filtering, and removal of chimera, undesirable sequences, and singletons 909,312 ITS region sequences were further rarefied to 28,812 sequences in each library with a total of 576,240 sequences (Table S19). These sequences were assigned to 5,028 OTUs at 97% sequence similarity.

We also compared the observed and expected OTU distribution to detect habitat generalist and specialist groups in the fungal community. Among the rarefied 5,028 OTUs in the whole fungal community, 446 habitat generalist OTUs and 97 habitat specialist OTUs were identified across all samples that represented 9% and 44.2% of the total sequences, respectively. The total fungal community associated with at least one sample from the two investigated sponge species was assigned to 242 OTUs (4.8% of the total dataset), of which 186 habitat generalists OTUs (8% of assigned sponge OTUs) and 56 habitat specialists OTUs (2.4% of the assigned sponge OTUs) from *Aplysina* species were represented. Because of the low representativeness of habitat generalist and specialist groups across sponge species, we only showed their community composition at the lower taxonomic level possible (Table S20a, b, respectively).

***Fungal alpha ecological metrics***

The rarefaction curves showed that none of the categories reached the plateau (Figure S9a). All investigated alpha ecological metrics followed the same pattern of results, with highest values observed in seawater, followed by *A. caissara, A. fulva*, and sediment samples (Table S21). Mean pairwise comparisons of observed OTUs richness index, *H’*, and *D_2_* indices revealed a significant difference between seawater and all other samples (*p* <0.05) (Table S22a). Whereas, mean pairwise comparisons of CHAO and ACE richness indices and Pielou’s evenness revealed a significant difference between seawater and samples from *A. fulva* and sediment (*p* <0.01) (Table S22a). Within these comparisons, samples from *A. caissara* revealed no significant difference (*p* >0.05) with all other samples (Table S22a).

The analysis of phylogenetic signal revealed no significant difference when comparing sponge species evolutionary inference with diversity indices of *H’* (*p* =0.229) and *D_2_* (*p* =0.3). These results suggest that a random distribution of the data has higher value for the goodness-of-fits than the observed sponge species relatedness.

***Fungal beta ecological metrics***

The nMDS ordination fungal community of whole and habitat specialist groups also revealed three main clusters: (***i***) *Aplysina* species, (***ii***) sediment, and (***iii***) seawater (Figure S10a, c). In the whole fungal community, *Aplysina* species presented higher dissimilarity distribution, whereas environmental replicates were tightly grouped to each other in a single cluster (Figure S10a). The nMDS ordination of habitat generalist group was more erratically distributed, with replicates in each treatment largely dispersed across both axes (Figure S10b). In the habitat specialist group, all replicates in each treatment presented high dissimilarity distribution mainly across the y-axis (Figure S10c). Nevertheless, in all fungal community groups, replicates from *Aplysina* species were grouped on one side of the x-axis ordination and apart from environmental samples. Permutational multivariate analysis of variance (PERMANOVA) and ANOSIM further confirmed that the compositions of the whole and habitat generalist-specialist groups were significantly different (*p* <0.001) (Table S23).

***Community composition at high taxonomic ranks***

Overall, 10 fungal phyla were retrieved. Seawater encompassed nine fungal phyla followed by *A. fulva* and sediment with six phyla each, and *A. caissara* with five phyla (Figure S11a, Table S24a). The two *Aplysina* species comprised together seven phyla. For *A. caissara*, Ascomycota, Basidiomycota, and unclassified Fungi were the most dominant phyla. Ascomycota and Basidiomycota were the most abundant phyla recovered from *A. fulva*. For seawater, Basidiomycota, Ascomycota, and unclassified Fungi were the dominant phyla. Unclassified Fungi dominated the sediment. The habitat generalist group of *A. caissara* was dominated by unclassified Fungi, while the habitat specialist group was dominated by Ascomycota, Mortierellomycota, and Basidiomycota (Figure S12a, c). Whereas for *A. fulva* Basidiomycota was the most abundant in the habitat generalist and specialist groups (Figure S12a, c).

In total, 31 fungal classes were detected across categories. The most diverse community composition was detected in seawater with 27 classes, followed by *A. fulva*, sediment, and *A. caissara* with 21, 20, and 18 classes, respectively (Figure 11b, Table S24b). The two sponge species together encompassed 23 classes. The classes unclassified Fungi, Agaricomycetes, Sordariomycetes, and Dothideomycetes were the most abundant associated with *A. caissara*. For *A. fulva*, the most dominant classes were Agaricomycetes, Tremellomycetes, unclassified Ascomycota, and Sordariomycetes (Figure 11b). For seawater the most dominant classes were Agaricomycetes, unclassified Fungi, and Dothideomycetes. For sediment, unclassified Fungi was the dominant class. For habitat generalists, *A. caissara* was dominated by unclassified Fungi and Sordariomycetes, and *A. fulva* by Tremellomycetes, unclassified Ascomycota, and Mortierellomycetes (Figure S12b). For habitat specialists, Dacrymycetes and Wallemiomycetes were the most abundant associated with *A. caissara* and unclassified Basidiomycota and Wallemiomycetes were the most abundant associated with *A. fulva* (Figure S12d).

To get insights regarding the unclassified lineages associated with the *Aplysina* species, further analysis was carried out at the OTU level. From a total 2,306 OTUs associated with *Aplysina* species 1,335 (57.9%) were unclassified at some level of the taxonomy affiliation, representing putative novel species. Indeed, 79.8% of the investigated OTUs presented similarity below 97% against NCBI ITS RefSeq Targeted Loci (RTL) database, suggesting that sponge species might be a tremendous untapped resource of microorganisms. Only 1.8% of the unclassified OTUs did not fullfil our blastn requirements resulting in no matches against the Silva database (Table S25a, b).

***Assemblage of fungal community***

The MST analysis performed with whole fungal community revealed that all samples were driven by deterministic assembly processes (MST <38%; Table S26a). Among sponge species, *A. fulva* exhibited the lowest stochasticity ratio (18%). None of the investigated samples in the whole fungal community fitted to the Sloan neutral community model prediction (Table S26b), supporting the predominance of deterministic processes shaping the fungal assemblage in the sponge species. For the habitat generalist-specialist groups, samples from both *Aplysina* species and sediment were driven mainly by deterministic assembly processes (MST <40% and <13% for habitat generalist and specialist groups, respectively; Table S26a), whereas samples from seawater were slightly driven by stochasticity processes (MST = 72% and 52% for habitat generalist and specialist groups, respectively). Nevertheless, none of the samples from the habitat generalist fungal group fitted the Sloan neutral community model prediction, due to a larger fit of the neutral model by randomly sampling of the source metacommunity (*R^2^*.pois) than the fit observed within the sample (*R^2^*), suggesting the predominance of deterministic processes shaping the prokaryotic assemblage in the seawater samples (Table S26b). On the other hand, seawater samples from the habitat specialist fungal group fitted the Sloan neutral community model prediction but with low fit *R^2^* value (*R^2^* = 0.04) (Table S26b).

***Specificities and commonalities: shared and exclusive OTUs***

The Venn diagram showed that from a total of 5,323 OTUs, 4,541 were exclusively observed in the categories and only 13 were common to all categories (Figure S13a, Table 27). For *A. caissara*, 76.2% of its fungal community was specific of this host sponge and the most abundant classes were affiliated to Agaricomycetes, Dothideomycetes, Tremellomycetes, and unclassified Fungi (Table S27a). For *A. fulva*, 74.5% of its fungal community was host-specific, and the most dominant classes were affiliated to Agaricomycetes, unclassified Fungi, and Eurotiomycetes (Table S27b). Around 10% of the OTUs were common to both *Aplysina* species, the most dominant were Agaricomycetes, unclassified Fungi, and Dothideomycetes (Table S27c). For seawater, 89.6% of its fungal community was specific of this category and the most abundant classes were affiliated to Agaricomycetes, unclassified Fungi, unclassified Ascomycota, Sordariomycetes, and Eurotiomycetes (Table S27d). For sediment, 79.8% of its fungal community was registered in this category. The class unclassified Fungi and Agaricomycetes were the most abundant in sediment (Table S27e). The core encompassed only 13 OTUs (0.26%) that were affiliated to the class Tremellomycetes and Agaricomycetes (Table S27f).

Thirty-two OTUs were common to sponge species and seawater samples. From these, five were enriched in seawater (two unclassified Fungi, unclassified Eurotiales, and unclassified Agaricomycetes) and one enriched in *A. caissara* and seawater (*Rhodotorula mucilaginosa*).

The habitat generalist group is composed by two phyla and 16 OTUs were common to more than one category (Figure S14). The habitat specialist group encompassed only one phylum (Basidiomycota) and four OTUs were shared by more than one category (Figure S15).

***Linear discriminant analysis effect size (LEfSe) analysis***

From all taxonomic affiliations classified until class level, only eight could explain the variability identified in the fungal community associated with sponge species and detected in environmental samples (Figure S13c, Table S28a). The lineages for *A. caissara* were affiliated to Ascomycota, Sordariomycetes, and Microbotryomycetes. Seawater was represented by Basidiomycota, Agaricomycetes, and Dothideomycetes; and sediment by unclassified Fungi and Chytridiomycota. Only one lineage (unclassified Fungi) was significantly enriched among habitat generalist group associated with *A. caissara* (Table S28b). The habitat specialist group also had only one lineage (Sordariomycetes) enriched associated with *A. caissara* (Table S28c).

**Unicellular eukaryotic dataset**

All samples from *A. fulva* and two from *A. caissara* showed low abundance in the unicellular eukaryotic dataset and therefore, these samples were removed to enhance sequence depth of the overall data. By removing these samples, the original dataset with 561 OTUs (presented in the main text) was largely improved and the new dataset encompassed by 1,479 OTUs. Indeed, a total, 2,790,422 V9-region 18S rRNA gene sequences were obtained. After quality filtering, removal of chimera, undesirable sequences, including eukaryotic sequences other than the unicellular ones, and singletons, 884,465 sequences were rarefied to the same library depth of 700 sequences, resulting in 12,600 sequences that were assigned to 1,479 OTUs at 97% sequence similarity (Table S29).

By comparing the observed and expected OTU distribution we were able to detect habitat generalist and specialist groups in the unicellular eukaryotic community. A total of nine OTUs (0.36% of the total sequences) were assigned as habitat generalist and all of them were exclusively detected in sponge species. Other 36 OTUs were assigned as habitat specialist across all samples and represented 41.7% of the total sequences. When we considered only those associated with at least one sample from the two sponge species, a total of 28 OTUs (1.9% of the total dataset) was encountered, of which nine were assigned as habitat generalist OTUs and 19 as habitat specialist OTUs. These represented 1.7% and 3.7% of assigned sponge OTUs, respectively. Once again, because of the low representativeness of habitat generalist and specialist groups across sponge species, we only showed their community composition at lower taxonomic level possible without further community analyses (Table S30b, c, respectively). In addition, samples from habitat generalist were not investigated in all analyses because of their extreme low representativeness across samples.

***Unicellular eukaryotic alpha ecological metrics***

The rarefaction curves demonstrated that none of the categories reached the plateau (Figure S9b). Once again, the results among the investigated richness indices were congruent. The highest observed OTUs, CHAO, and ACE richness indices were detected in sediment followed by seawater, *T. ignis*, and *A. caissara* (Table S21). Mean pairwise comparisons for each of these richness indices revealed significant differences (*p* <0.001) between sediment and all other categories (Table S22b). Samples from seawater also revealed significantly larger (*p* <0.05) richness indices than samples from sponge species (Table S22b). The results from *H’* and *D_2_* diversity and Pielou’s evenness indices ranged from the largest values encountered in sediment followed by *T. ignis,* seawater, and *A. caissara* (Table S21). Mean pairwise comparisons revealed significant dissimilarities (*p* <0.01) for *H’* and *D_2_* diversity and Pielou’s evenness indices between sediment and all other categories (Table S22b).

For the unicellular eukaryotic community, a slight significant difference (*p* =0.0425) was observed when comparing host evolutionary inference with *D_2_* diversity index, but not for *H’* diversity index (*p* =0.071). These results, although slightly conflicted, might be explained by the algorithm used to calculate both diversity indices. Inverse Simpson’s index is less sensitive to the difference in taxa richness than Shannon's index.

***Unicellular eukaryotic beta ecological metrics***

The nMDS ordination of whole unicellular Eukaryotic and habitat specialist groups revealed four clusters, one for each category (Figure S10d, e). Replicates from seawater formed a concise cluster, whereas the other categories presented a more dissimilar pattern, with samples from the same category apart from each other, especially for *T. ignis*. Permutational multivariate analysis of variance (PERMANOVA) and ANOSIM further confirmed that the compositions of the whole and habitat specialist groups were significantly different (*p* <0.001) with high fit R^2^ value (Table S23).

***Community composition at high taxonomic ranks***

In total, 27 microbial eukaryotic phyla were recovered. Seawater encompassed the most diverse community composition with 20 phyla, followed by sediment, *A. caissara*, and *T. ignis* with 19, 5, and 14 phyla, respectively. Together, the two sponge species comprised 18 phyla (Figure S16a, Table S31a). Unclassified Eukaryota was the most abundant phylum associated with the two sponge species. The dominant identifiable phyla in *A. caissara* and *T. ignis* were Florideophycidae and Diatomea, respectively. Diatomea and unclassified Eukaryota were the most dominant phyla detected in seawater. For sediment, the most abundant phyla were Diatomea, Dinoflagellata, and unclassified Eukaryota. The habitat generalist group was dominated by unclassified Eukaryota, whereas the habitat specialist group, *A. caissara* and *T. ignis* were dominated by Chlorophyta and Ochrophyta, respectively (Figure S17a).

In total, 64 classes were observed among samples. The most diverse community composition was detected in sediment with 40 classes, followed by seawater, *T. ignis*, and *A. caissara* with 39, 29, and 27, respectively (Table S16b, Table S31b). *A. caissara* and *T. ignis* together encompassed 38 classes. Unclassified Eukaryota was the most abundant class associated with the two sponge species. Nemaliophycidae and Mediophyceae were the second most abundant class in *A. caissara* and *T. ignis*, respectively. For seawater, the most dominant classes were Bacillariophyceae and unclassified Eukaryota. For sediment, Mediophyceae and unclassified Diatomea were the most dominant classes. The habitat generalist group for both sponge species was dominated by unclassified Eukaryota, whereas for habitat specialist, *A. caissara* and *T. ignis* were largely dominated by Nemaliophycidae and unclassified Eukaryota, respectively (Figure S17b).

To further explore the unclassified lineages associated with *A. caissara* and *T. ignis*, analysis at the OTU level was performed. Overall, from a total of 515 OTUs affiliated to sponge species 386 (74.9%) were unclassified at some level of the taxonomy affiliation. Indeed, 61.4% of the unclassified OTUs showed similarity below 97% against the Silva database, suggesting once again an important untapped resource of microorganisms. A total of 16.6% of the unclassified OTUs did not fullfil our BLASTn requirements resulting in no matches against the Silva database (Table S32a-b).

***Assemblage of unicellular eukaryotic community***

The MST analysis performed with whole unicellular eukaryotic community revealed that samples from *A. caissara,* sediment, and seawater were driven by deterministic assembly processes (MST < 50%) (Table S33a). The community assembly of unicellular eukaryotes from *T. ignis* was mainly driven by stochasticity processes (MST = 63%). For habitat specialist group, samples from *T. ignis* and sediment were mainly driven by stochasticity processes (MST > 50%). Once again, samples from *A. caissara* and seawater exhibited stochasticity ratio bellow 50%, supporting the predominance of deterministic assembly processes (Table S33a). None of the whole unicellular eukaryotic communities in the samples fitted to the Sloan neutral model prediction (Table S33b). However, the Sloan neutral model fitted to the habitat specialist group associated with *T. ignis* (*R^2^* = 0.27), sediment (*R^2^* = 0.52), and seawater (*R^2^* = 0.98) samples. The estimated migration rate (*m*) of habitat specialists was larger for seawater samples (*m =* 0.16) than for *T. ignis* (*m =* 0.002), which suggested higher dispersion limitation within sponge habitat specialist group than seawater samples (Table S33b).

***Specificities and commonalities: shared and exclusive OTUs***

The Venn diagram revealed that 1,376 from a total of 1,479 OTUs were specific to each category and six were common to all categories (Figure S13b, Table S34). Overall, the unclassified Eukaryota was the most abundant lineage associated with *A. caissara* and *T. ignis* (Table S34a-b). For *A. caissara*, 65.5% of its unicellular eukaryotic was host-specific and the second most abundant OTU was affiliated to Syndiniales (Table S34a). For *T. ignis*, 82.7% of its unicellular eukaryote was species specific, and among the identifiable classes Corallinophycidae, Bacillariophyceae, and Mediophyceae were the most abundant (Table S34b). Thirteen OTUs were commonly detected across all sponge species and the most abundant were unclassified Eukaryota and Nemaliophycidae (Table S34c). For seawater, 93% of the unicellular eukaryotic was specific to it and unclassified Eukaryota and Bacillariophyceae were the most dominant classes (Table S34d). For sediment, 83.4% of its unicellular eukaryotic was specifically detected in this category and the most abundant classes were unclassified Eukaryota, Dinophyceae, and Intramacronucleata (Table S34e). The most abundant classes in the core were Mediophyceae, unclassified Diatomea and Bacillariophyceae (Table S33f). As observed in the fungal community, all investigated sponge species and environmental samples revealed distinct community composition, suggesting that each is a unique environment harboring their own suitable community.

Only five OTUs were shared between sponges and seawater. From these, only OTU81 (unclassified Diatomea) was enriched in seawater.

The habitat generalist group is composed by only one phylum (unclassified Eukaryota) and no phylogenetic related OTUs was common to more than one category. The habitat specialist group encompassed 10 abundant phyla and similar to the habitat generalist, the habitat specialist group had no phylogenetic related OTUs detected in more than one category.

***Linear discriminant analysis effect size (LEfSe) analysis***

Only 22 lineages from all taxonomic affiliations classified could explain the variability observed in the unicellular eukaryotic community associated with sponge species and detected in environmental samples. Sediment and seawater had seven lineages each, followed by *T. ignis* and *A. caissara* with four each (Figure S13d, Table S35a). Each sponge species and environmental samples have their own distinct enriched lineages. No lineage was enriched in the habitat generalist and only one lineage (unclassified Eukaryota) was significantly enriched among habitat specialist group associated with *T. ignis* (Table S35b).

**Reference List**

Abarenkov, K.; Zirk, A.; Piirmann, T.; Pöhönen, R.; Ivanov, F.; Nilsson, R.H.; Kõljalg, U. UNITE Mothur Release for Fungi. Version04.02.2020. UNITE Community. 2020. Available online: https://plutof.ut.ee/#/doi/10.15156/BIO/786381 (accessed on 11 December 2020).

Amaral-Zettler, L.A., McCliment, E.A., Ducklow, H.W., and Huse, S.M. (2009). A method for studying protistan diversity using massively parallel sequencing of V9 hypervariable regions of small-subunit ribosomal RNA Genes. *PLoS One*. 4, e6372. doi:10.1371/journal.pone.0006372.

Apprill, A., Mcnally, S., Parsons, R., and Weber, L. (2015). Minor revision to V4 region SSU rRNA 806R gene primer greatly increases detection of SAR11 bacterioplankton. *Aquat. Microb. Ecol.* 75, 129-137. doi:10.3354/ame01753.

Edgar, R.C., Haas, B.J., Clemente, J.C., Quince, C., and Knight, R. (2011). UCHIME improves sensitivity and speed of chimera detection. *Bioinformatics* 27, 2194–2200. doi:10.1093/bioinformatics/btr381.

Gardes, M., and Bruns, T.D. (1993). ITS primers with enhanced specificity for basidiomycetes-application to the identification of mycorrhizae and rusts. *Mol Ecol*. 2,

113-118.

Parada, A.E., Needham, D.M., and Fuhrman, J.A. (2016). Every base matters: Assessing small subunit rRNA primers for marine microbiomes with mock communities, time series and global field samples. *Environ. Microbiol.* 18, 1403–1414. doi:10.1111/1462-2920.13023.

Quast, C., Pruesse, E., Yilmaz, P., Gerken, J., Schweer, T., Yarza, P., et al. (2013). The SILVA ribosomal RNA gene database project: Improved data processing and web-based tools. *Nucleic Acids Res.* 41, D590-D596. doi:10.1093/nar/gks1219.

Schloss, P.D., Westcott, S.L., Ryabin, T., Hall, J.R., Hartmann, M., Hollister, E.B., et al. (2009). Introducing mothur: Open-source, platform-independent, community-supported software for describing and comparing microbial communities. *Appl. Environ. Microbiol.* 75, 7537–7541. doi:10.1128/AEM.01541-09.

Smith, D.P., and Peay, K.G. (2014). Sequence depth, not PCR replication, improves ecological inference from next generation DNA sequencing. *PLoS One* 9. doi:10.1371/journal.pone.0090234.

Stoeck, T., Bass, D., Nebel, M., Christen, R., Jones, M.D.M., Breiner, H.W., et al. (2010). Multiple marker parallel tag environmental DNA sequencing reveals a highly complex eukaryotic community in marine anoxic water. *Mol. Ecol.* 19, 21–31. doi:10.1111/j.1365-294X.2009.04480.x.

Yilmaz, P., Parfrey, L.W., Yarza, P., Gerken, J., Pruesse, E., Quast, C., et al. (2014). The SILVA and “all-species Living Tree Project (LTP)” taxonomic frameworks. *Nucleic Acids Res.* 42, 643–648. doi:10.1093/nar/gkt1209.
